# Tumour-Secreted Protein S (ProS1) Activates a Tyro3-Erk Signalling Axis and Protects Cancer Cells from Apoptosis

**DOI:** 10.3390/cancers11121843

**Published:** 2019-11-22

**Authors:** Nour Al Kafri, Sassan Hafizi

**Affiliations:** School of Pharmacy and Biomedical Sciences, University of Portsmouth, St. Michael’s Building, White Swan Road, Portsmouth PO1 2DT, UK

**Keywords:** Tyro3, Gas6, growth factors, receptor tyrosine kinase, protein S, cell survival, signal transduction, vitamin K, paracrine

## Abstract

The TAM subfamily (Tyro3, Axl, MerTK) of receptor tyrosine kinases are implicated in several cancers, where they have been shown to support primary tumorigenesis as well as secondary resistance to cancer therapies. Relatively little is known about the oncogenic role of Tyro3, including its ligand selectivity and signalling in cancer cells. Tyro3 showed widespread protein and mRNA expression in a variety of human cancer cell lines. In SCC-25 head and neck cancer cells expressing both Tyro3 and Axl, Western blotting showed that both natural TAM ligands ProS1 and Gas6 rapidly stimulated Tyro3 and Erk kinase phosphorylation, with ProS1 eliciting a greater effect. In contrast, Gas6 was the sole stimulator of Axl and Akt kinase phosphorylation. In MGH-U3 bladder cancer cells, which express Tyro3 alone, ProS1 was again the stronger stimulator of Tyro3 and Erk stimulation but additionally stimulated Akt phosphorylation. Conditioned medium from ProS1-secreting 786-0 kidney cancer cells replicated the kinase activation effects of recombinant ProS1 in SCC-25 cells, with specificity confirmed by ProS1 ligand traps and warfarin. In addition, ProS1 protected cancer cells from acute apoptosis induced by staurosporine, as well as additionally, long-term serum starvation-induced apoptosis in MGH-U3 cells (Tyro3 only), which reflects its additional coupling to Akt signalling in these cells. In conclusion, we have shown that ProS1 is a tumour-derived functional ligand for Tyro3 that supports cancer cell survival. Furthermore, the ProS1-Tyro3 interaction is primarily coupled to Erk signalling although it displays signalling diversity dependent upon its representative expression as a TAM receptor in tumour cells.

## 1. Introduction

The TAM (Tyro3, Axl, MerTK) subfamily of transmembrane receptor tyrosine kinases (RTKs) has been shown to regulate normal physiological processes such as tissue homeostasis and innate immunity [1]. Consequently, dysregulation of TAM signalling has been associated with chronic inflammatory and autoimmune conditions [2,3,4]. In addition, TAM signalling has been implicated in cancers, where each TAM RTK has in different studies been shown to regulate cancer cell growth, survival, proliferation, differentiation, migration or invasion [1,5]. The TAMs are also increasingly being implicated as mediators of chemoresistance to other targeted therapies such as anti-EGFR (epidermal growth factor receptor) agents [6]. Therefore, the TAMs present as attractive new targets in therapies aimed at combating tumour growth and spread. 

The protein structures of TAM receptors are identifiable by two N-terminal immunoglobulin (Ig)-like domains and two fibronectin type III domains in the extracellular region, a single-pass trans-membrane domain, and an intracellular domain with intrinsic tyrosine kinase activity, which also acts as a binding site for other signal molecules [7]. A further distinguishing feature for the TAM subfamily from other RTKs is the presence of a conserved KW(I/L)A(I/L)ES sequence in the kinase domain [8]. The human Tyro3 protein contains 890 amino acids and the molecular weight ranges between 120–140 kDa due to post-translational modifications, including glycosylation [1,9]. 

The natural ligands for the TAMs are two homologous vitamin K-dependent proteins: Gas6, which binds all three TAMs with binding affinity in the order Axl > Tyro3 > MerTK [10], and the coagulation regulator protein S (ProS1), which generally is believed to bind Tyro3 and MerTK but not Axl [11,12]. ProS1 and Gas6 are structurally similar in their compositions of a series of γ-carboxyglutamic acid (Gla) residues at the N-terminal, a loop region, four EGF-like repeats, and a sex hormone-binding globulin (SHBG)-like structure at the C-terminal, which is composed of two globular laminin G-like (LG) domains with calcium-binding sites [11,13]. The modified Gla residues within the N-terminal of the protein are derived from post-translational modification of glutamic acid residues in a vitamin K-dependent manner, and this region, known as the Gla domain, has significant binding affinity for phosphatidylserine-bearing membranes, as are present on activated platelets or apoptotic cells [4,14,15]. The Gla domain mediates this membrane interaction whereas the SHBG domain binds to the Ig domains of the TAM receptors and mediates receptor dimerization and activation. As a distinguishing feature from Gas6, ProS1 contains a thrombin-sensitive cleavage sequence within the N-terminal loop region, which enables its role as a major negative regulator of the blood coagulation cascade [16,17]. ProS1 in plasma is mainly synthesized and secreted by liver hepatocytes, which underscores this role in blood coagulation regulation. In addition, alternative cellular sources of ProS1 have also been identified, including endothelial cells, macrophages, dendritic cells and T cells, vascular smooth muscle cells, and glial cells [14]. However, relatively little is currently known about the role of ProS1 as a TAM ligand for tumours, with a few studies so far having reported ProS1 overexpression in different cancers [18].

Our lab has previously provided critical insights into the role of Axl and the phenomenon of EGFR-Axl hetero-dimerisation in cancer cells, as well as the regulation of cell invasion by EGFR through utilising Axl signalling pathways [19]. However, relatively little is known about Tyro3 in terms of its cancer expression profile, mechanisms of activation and cellular signalling and functional roles. Insights into the cellular and physiological functions of Tyro3 in different bodily systems have been provided from studies on the CNS [20], platelet aggregation [21], osteoclastic bone resorption [22] and reproductive system development [23]. In cancer, some recent studies have shown Tyro3 to be strongly expressed in cancer cell lines from melanoma, ovarian, hepatocellular, thyroid, colon and bladder cancers [24]. Nevertheless, there is a dearth of knowledge on the mechanisms of activation of Tyro3, especially that involving the ligand–receptor interaction. It is widely believed that Gas6 has, as a TAM ligand, a greater affinity towards Axl as compared to Tyro3 [2]. However, the affinities of both ligands for Tyro3 are greatly enhanced in the presence of phosphatidylserine in the membrane, with this effect being greater for Tyro3/MerTK than for Axl. While many signalling pathways have been well-characterized downstream of MerTK and Axl, few studies have directly addressed signalling pathways downstream of Tyro3. For example, Tyro3 has been linked to Akt in hepatocellular carcinoma, to regulate cell survival [25,26] and Erk signalling to regulate proliferation in breast cancer [27]. Here, we have focused on the specific role of Tyro3 expression and activation in cancers and attempted to delineate the downstream signalling pathways activated by ProS1-Tyro3 as compared to Gas6-Axl. 

In the present study, we have investigated the expression of Tyro3 in a variety of human cancer cell lines and revealed that the ligand selectivity of Tyro3 is a factor of TAM expression profile in cancer cells. Furthermore, we have investigated the signalling pathways activated specifically via Tyro3 and the functional effects of Tyro3 signalling in cancer cells. Moreover, we have identified cancer cells as a source of functional ProS1 as a potential paracrine or autocrine Tyro3 ligand. These novel data shed further light on the currently poorly understood roles of both Tyro3 and ProS1 in tumorigenesis and highlight Tyro3 as a novel cancer therapeutic target in addition to Axl.

## 2. Results

### 2.1. Expression of TAM Receptors and TAM Ligands in Human Cancer Cell Lines

**Figure 1:**
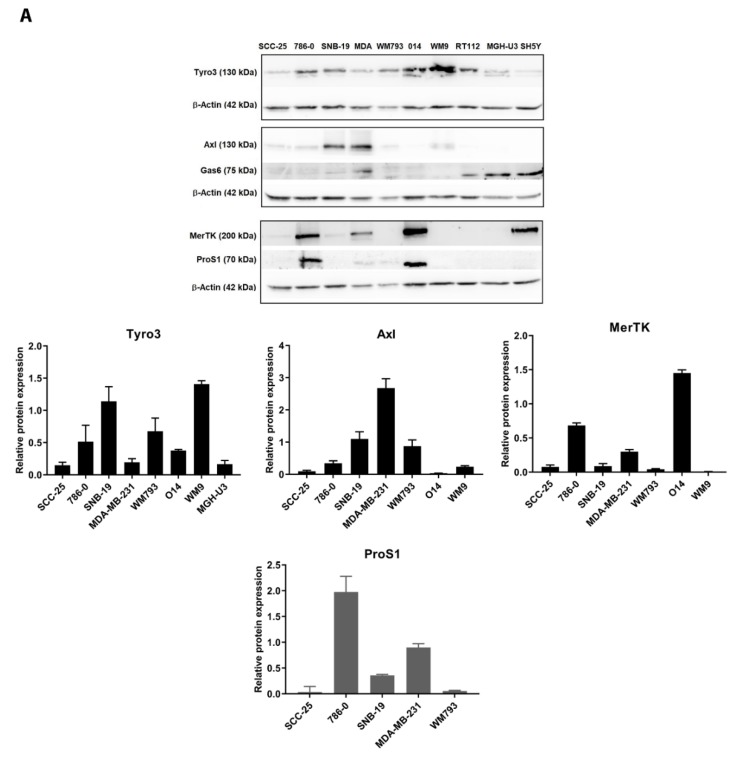

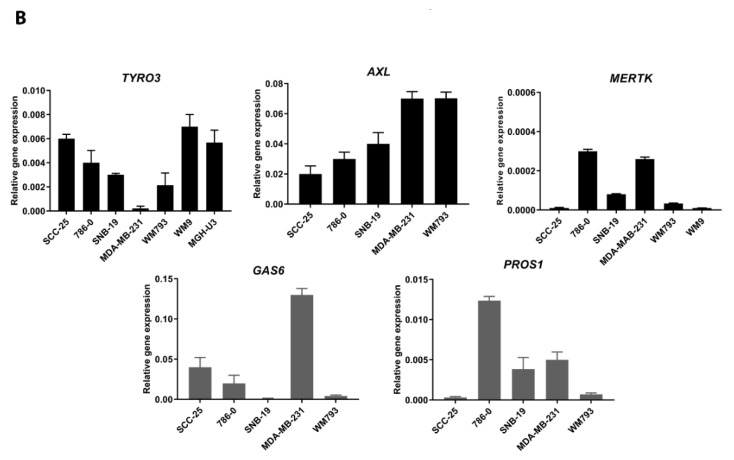
Expression of TAM RTKs and their ligands in a panel of human cancer cell lines (details in Supplementary Table S1). (**A**) Representative Western blot image from analysis of TAM receptors in protein extracts from various cultured human cancer cell lines, with accompanying graphs of protein quantification by densitometry. Bars are mean ± SEM protein expression normalized against β-actin as loading control protein (n = 3–6 separate experiments). (**B**) Quantitative RT-PCR analysis of mRNA expression of the genes for Axl, MerTK, Tyro3, Gas6 and ProS1 in extracts from five human cancer cell lines. Bars are mean ± SEM relative gene expression normalized against housekeeping gene *GAPDH* (n = three separate experiments).

Western blot screening of TAM receptors and ligands was performed on protein extracts from 10 cell lines from a variety human cancers (cell line list in Supplementary Table S1). At protein level, Tyro3 was the most ubiquitously expressed of the three TAM receptors in the cancer cell lines tested (Figure 1). Quantitation from multiple Western blot results showed the expression of Tyro3 to be highest in melanoma, glioma and kidney cancer cells (Figure 1A). In comparison, the expression profiles of the other TAM receptors Axl and MerTK were more discrete, with strong expression of Axl observed in breast cancer and glioma cells, whilst MerTK was most highly expressed in melanoma and kidney cancer cells. With the TAM ligands, ProS1 was found to be expressed highly in 786-0 kidney cancer cells and in one melanoma cell line (Figure 1A). 

The protein expression patterns of TAM receptors and ligands in human cancer cells were largely mirrored at the mRNA expression level as observed by RT-qPCR analysis (Figure 1B). Tyro3 also showed the most widespread mRNA expression whilst Axl and MerTK expression patterns were more discrete. In addition to ProS1, Gas6 was also found to be strongly expressed in particular cancer cell types, with the highest levels in MDA-MB-231 breast cancer cells. Therefore, certain tumour cells express TAM ligands in addition to TAM receptors, indicating the potential for autocrine or paracrine regulation. 

### 2.2. ProS1 Is a Preferential Ligand for Tyro3 than Gas6

**Figure 2:**
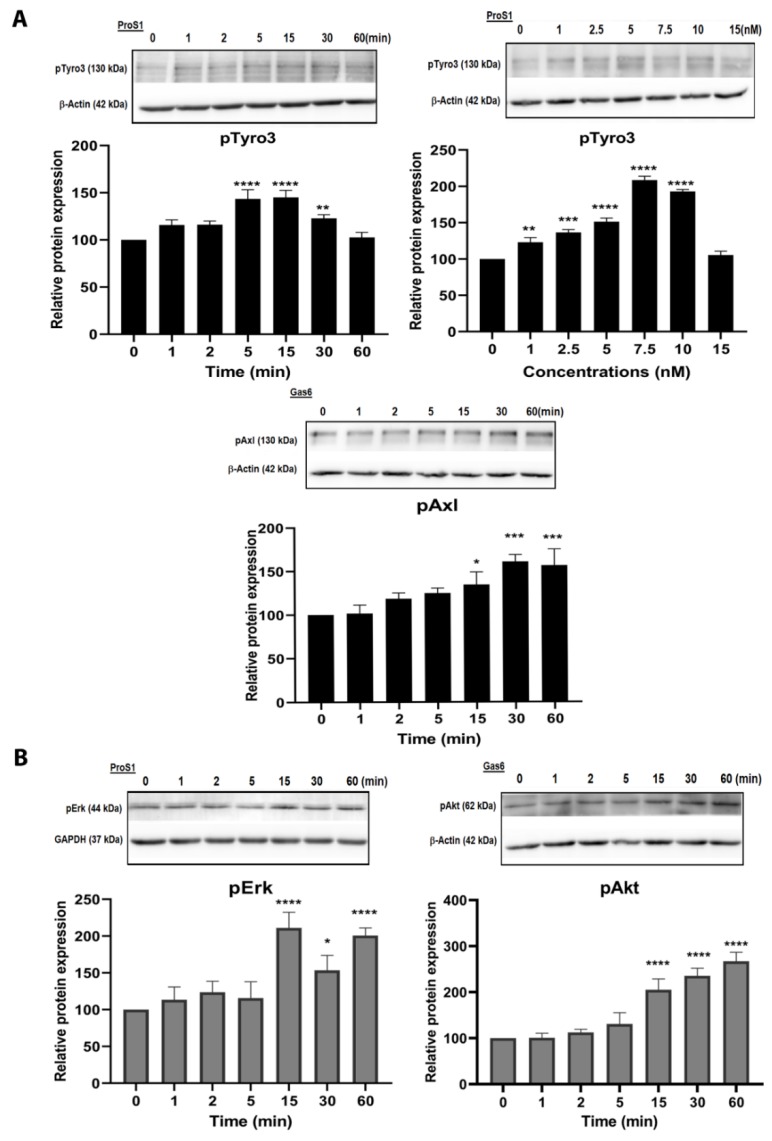
Effect of ProS1 and Gas6 stimulation on phosphorylation of TAM receptors and intracellular signalling kinases in SCC-25 cells. (**A**) Representative Western blots showing phosphorylated Tyro3 (pTyro3) protein in SCC-25 cells stimulated by ProS1 (7.5 nM) in time-course and dose response experiments, and phosphorylated Axl (pAxl) protein in cells stimulated over a time-course by Gas6 (5.7 nM). (**B**) Representative Western blot images show time-course of Erk phosphorylation (pErk) and Akt phosphorylation (pAkt). Accompanying graphs show protein quantification by densitometric analysis of bands. Data are mean ± SEM protein expression normalized against GAPDH or β-actin as loading control protein; ANOVA with Tukey’s multiple comparison post-hoc analysis; **** *p* < 0.0001, *** *p* < 0.001, ** *p* < 0.01, * *p* < 0.05, versus control (time 0 or untreated) (n = three separate experiments).

**Figure 3:**
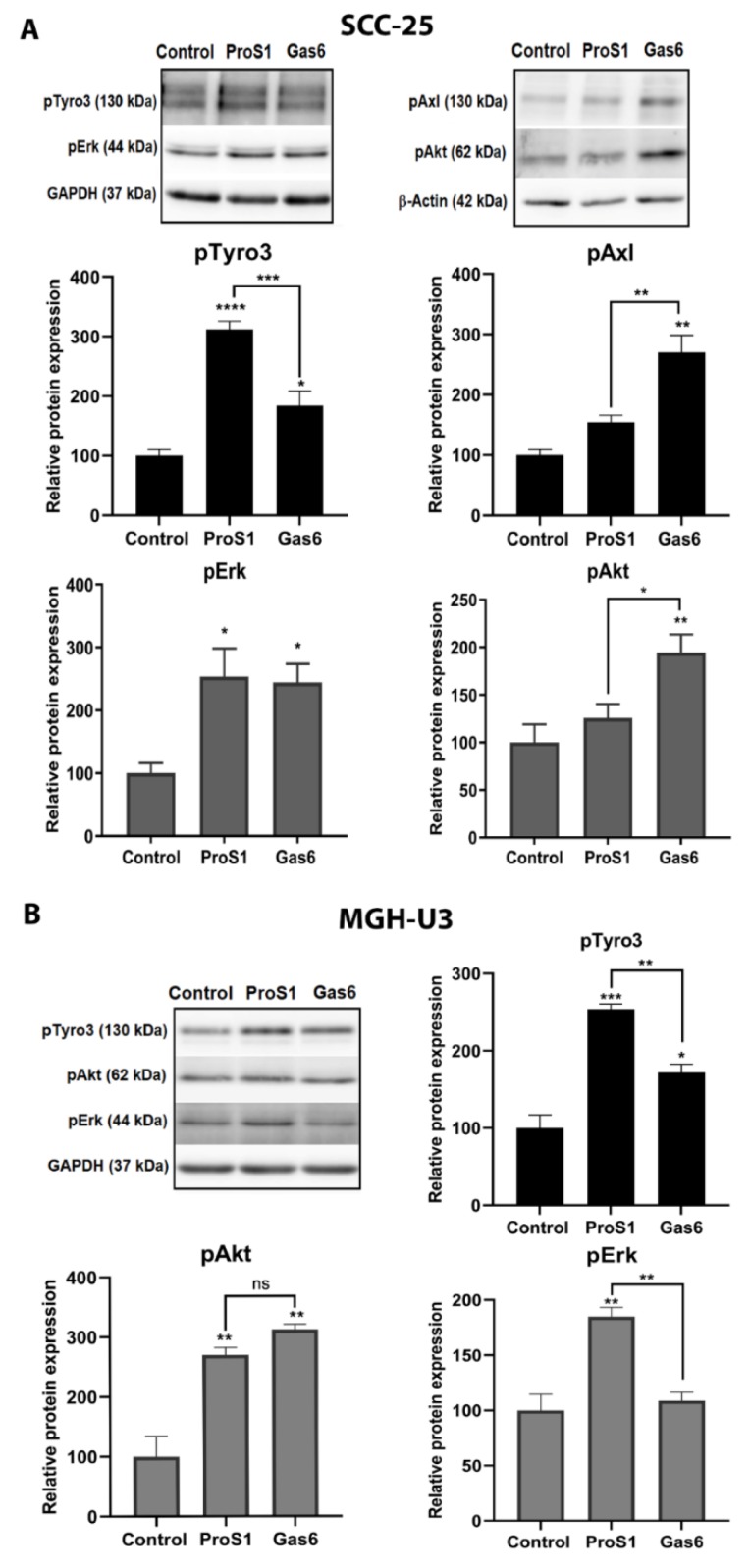
Role of TAM receptor expression profile in mediating the effects of ProS1 and Gas6 on RTK and intracellular signalling kinase phosphorylation. Experiments were conducted on cancer cell lines SCC-25 (express Tyro3 and Axl) and MGH-U3 cells (express Tyro3 only). Representative Western blot showing receptor activation and downstream signalling (Akt and Erk phosphorylation) by Gas6 and ProS1 in SCC-25 cells (**A**) and MGH-U3 cells (**B**) with accompanying graphs of densitometric quantification of bands. Data are mean ± SEM protein expression normalized against GAPDH as loading control protein; ANOVA with Tukey’s multiple comparison *post-hoc* analysis; **** *p* < 0.0001, *** *p* < 0.001, ** *p* < 0.01, * *p* < 0.05 and ns, not significant versus control or for comparisons indicated by lines (n= three or more separate experiments).

Having identified cancer cell lines with Tyro3 expression, we selected SCC-25 head and neck carcinoma cells for further study as these cells showed a consistent response to ligand stimulation (Supplementary Figures S1 and S2) and with less potential influence of the other TAM receptors. We determined the activation profile of Tyro3 in response to stimulation by exogenous recombinant TAM ligands in terms of phosphorylation of the receptor and associated intracellular signalling proteins. Western blots showed that ProS1 rapidly stimulated Tyro3 phosphorylation in SCC-25 cells, peaking at 5 min and decreasing from 15 min (Figure 2A). Significant Tyro3 activation was observed by ProS1 at 1nM concentration, with maximal activation occurring at 7.5 nM (Figure 2A). The same profile of Tyro3 activation by ProS1 was also observed in several of the other cancer cell lines expressing Tyro3 (Supplementary Figure S1A). According to these observations, ProS1 stimulation at 7.5 nM and for 9 min were selected for use in subsequent experiments. In contrast to ProS1, Gas6 was a weak stimulator of Tyro3 phosphorylation in SCC-25 cells (Figure 3A), whereas it strongly and rapidly stimulated Axl phosphorylation (Figure 2A and Figure 3A), which confirmed its primary role as a ligand for Axl [5]. 

Intracellular signalling downstream of receptor activation was next investigated in SCC-25 cells. ProS1 and Gas6 both activated intracellular signalling molecules downstream of their activation of the RTKs in different ways. ProS1 rapidly induced Erk kinase phosphorylation by approximately two-fold (Figure 2B and Supplementary Figure S2B), whereas Gas6 stimulated Akt kinase phosphorylation, a well-known downstream Axl target, in these and other cells (Figure 2B and Supplementary Figure S1B). 

### 2.3. In Cells Expressing Tyro3 as Sole TAM Receptor, the ProS1-Tyro3-Erk Signalling Axis Prevails but Is Diversified

Whilst the Gas6-Axl-Akt signalling axis has been observed several times, a discrete signalling pathway downstream of the ProS1-Tyro3 interaction remains to be characterised. Therefore, the effects of TAM ligands ProS1 and Gas6 were tested and compared in two distinct cell lines: SCC-25 cells which express both Tyro3 and Axl, and MGH-U3 cells which express Tyro3 as sole TAM receptor [28] (Figure 1). ProS1 stimulated Tyro3 and Erk phosphorylation in both cell lines (Figure 3), as well as Akt phosphorylation in MGH-U3 cells (Figure 3b). By comparison, Gas6 stimulated both Axl and (weakly) Tyro3, as well as Erk and Akt phosphorylation in SCC-25 cells (Figure 3A). In MGH-U3 cells, acting via Tyro3, Gas6 was able to stimulate Tyro3 and Akt but not Erk phosphorylation (Figure 3B). Therefore, these results show that Tyro3 is the main TAM receptor coupled to Erk signalling in cancer cells, but is also able to diversify its signalling pathway interactions, as well as ligand sensitivity, when present as the sole TAM receptor at a sufficient expression level in cancer cells. 

### 2.4. ProS1 Is Secreted from Cancer Cells as a Functionally Active Vitamin K-Dependent Ligand for Tyro3

**Figure 4:**
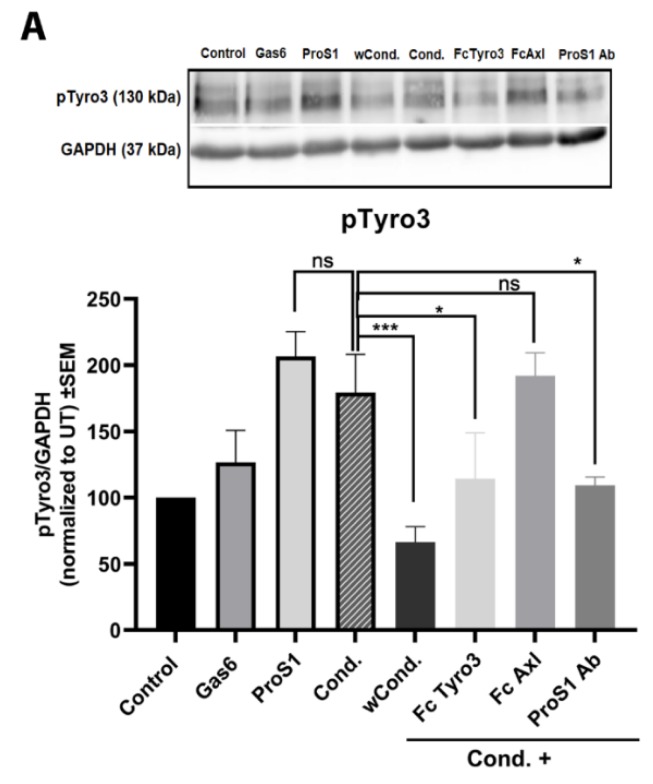

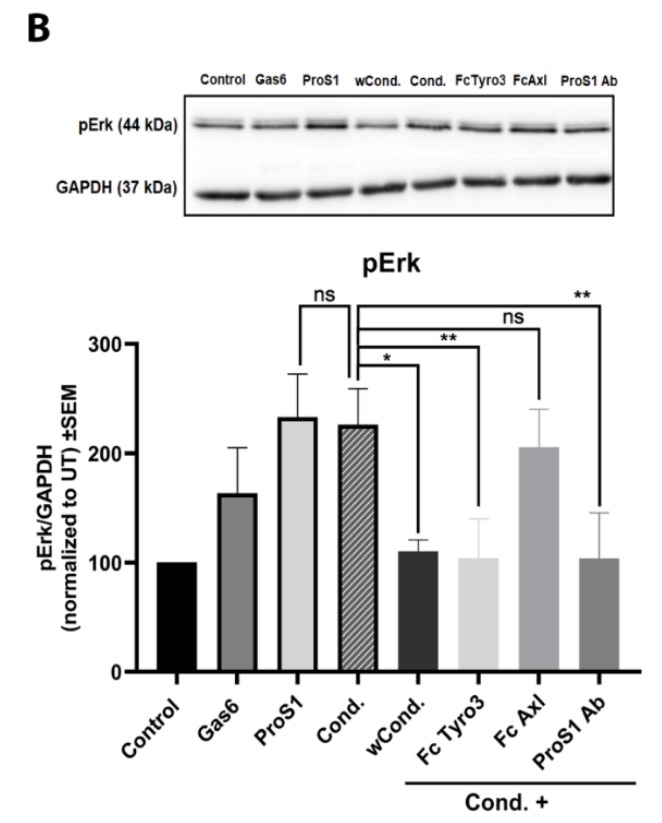
The effect of conditioned medium from 786-0 (producer) cancer cells on Tyro3 and Erk phosphorylation in SCC-25 (responder) cells. Cells were incubated for 9 min with recombinant proteins ProS1 (7.5 nM), Gas6 (5.7 nM), or conditioned media (cond.), without or with soluble TAM receptor ectodomains Fc-Axl or Fc-Tyro3 (3µg/mL), or anti-ProS1 antibody (5µg/mL). Conditioned medium was also applied from producer cells that had been pre-incubated with warfarin (wCond.). Cell lysates underwent Western blotting to detect phospho-Tyro3 (**A**) and phospho-Erk (**B**) proteins. Quantitative analysis by densitometric analysis of bands is shown graphically below each blot image. Data are mean±SEM protein expression normalized against GAPDH as loading control protein; ANOVA with Tukey’s multiple comparison post-hoc analysis; *** *p* < 0.001, ** *p* < 0.01, * *p* < 0.05, and ns, not significant versus control (untreated, UT) or for comparisons indicated by lines (n = three separate experiments).

Having shown that exogenous recombinant ProS1 activates Tyro3 and downstream Erk signalling in human cancer cells, we also investigated the effect of endogenous ProS1 secreted by human cancer cells as a functional Tyro3 ligand. Thus, we collected conditioned medium from 786-0 kidney cancer cells, which we had observed to express high levels of endogenous ProS1 (Figure 1), which was added to SCC-25 cells as a responder system in experiments. The 786-0 cell-conditioned medium was able to stimulate both Tyro3 and Erk phosphorylation to the same extent as exogenous recombinant ProS1 (Figure 4). This effect was verified as being due to secreted ProS1 being functional as a vitamin K-dependent protein, as conditioned medium from 786-0 producer cells pre-incubated with the vitamin K antagonist warfarin (wCond.) had no effect on Tyro3/Erk phosphorylation in the SCC-25 responder cells (Figure 4). Furthermore, the conditioned medium effect was blocked by co-incubation with two forms of ligand traps: soluble Tyro3 ectodomain and anti-ProS1 neutralising antibody, whilst soluble Axl ectodomain had no blocking effect. Therefore, these data show that a biologically active, vitamin K-dependent ProS1 is secreted by cancer cells which can activate Tyro3 in cancer cells in a paracrine or autocrine manner. 

### 2.5. ProS1-Tyro3 Protects Cancer Cells from Apoptosis

**Figure 5:**
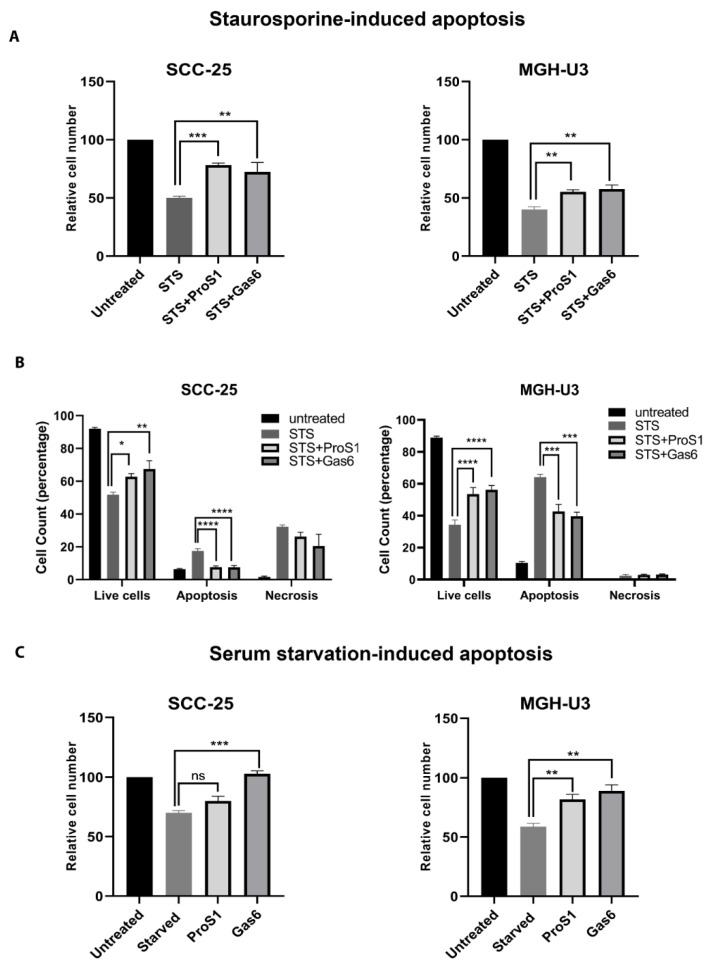
Effect of ProS1 and Gas6 on cancer cells undergoing apoptosis and role of TAM receptor expression profile in mediating these effects. MTS cell viability assay of SCC-25 (express Tyro3 and Axl) and MGH-U3 (express Tyro3 only) cells incubated for 20 h in the presence of staurosporine (0.1 µM) (short term) (**A**) or in serum-free medium for 20 days (long term) (**C**), with co-incubation with Gas6 or ProS1 added 1 h previously. (**B**) Flow cytometry results showing percentage of SCC-25 and MGH-U3 cells undergoing apoptosis (Annexin V-FITC/PI double-stained) by 20 h incubation with staurosporine (0.1 µM), with co-incubation with TAM ligands added 1 h previously. Graphs show proportions (%) of healthy, apoptotic and necrotic cells. Data are mean±SEM, and underwent ANOVA with Tukey’s multiple comparison *post-hoc* analysis; **** *p* < 0.0001, *** *p* < 0.001, ** *p* < 0.01, * *p* < 0.05 versus indicated control (no treatment or staurosporine-treated) or for comparisons indicated by lines (n = three experiments).

Having determined the receptor and signal pathway activation profile of ProS1 as a vitamin K-dependent TAM ligand, we investigated the functional effect of ProS1 on cells undergoing apoptosis under acute and prolonged conditions. In MTS assays, staurosporine (0.1 µM) was used to acutely trigger apoptosis, and the effects of TAM ligands on this was measured over 20 h. Under these conditions, both ProS1 and Gas6 significantly protected cells from apoptosis in both SCC-25 (Tyro3 and Axl) and MGH-U3 (Tyro3 only) cancer cell lines (Figure 5A). We confirmed that the staurosporine at 0.1 µM did not block Tyro3 activity in cells, nor did it prevent Tyro3 activation by ProS1 in it presence (Supplementary Figure S4). Flow cytometry experiments also revealed the protective effect of both ligands Gas6 and ProS1 on staurosporine-induced acute apoptosis (Figure 5B), where presence of the ligands reduced the percentage of cells undergoing apoptosis as compared to cells treated with staurosporine alone (Figure 5B and Supplementary Figure S3). We also observed the same protective effects of ProS1 and Gas6 on viability of both cancer cells lines using an alternative apoptosis-inducing agent, cisplatin (Supplementary Figure S5). Furthermore, under conditions of long-term serum starvation, Gas6 significantly conferred a survival effect in both cell lines, whereas ProS1 significantly protected only the MGH-U3 cells (express Tyro3 only) (Figure 5C). 

We also employed a MEK inhibitor compound, PD0325901, to demonstrate the involvement of the Erk signalling pathway in mediating the anti-apoptotic effect of ProS1 in MTS assays. Whilst not further influencing staurosporine-induced apoptosis of cells, PD0325901 was able to block the protective effect of ProS1 (Supplementary Figure S6). This confirms the functional role of the ProS1-Tyro3-Erk signalling axis. 

**Figure 6:**
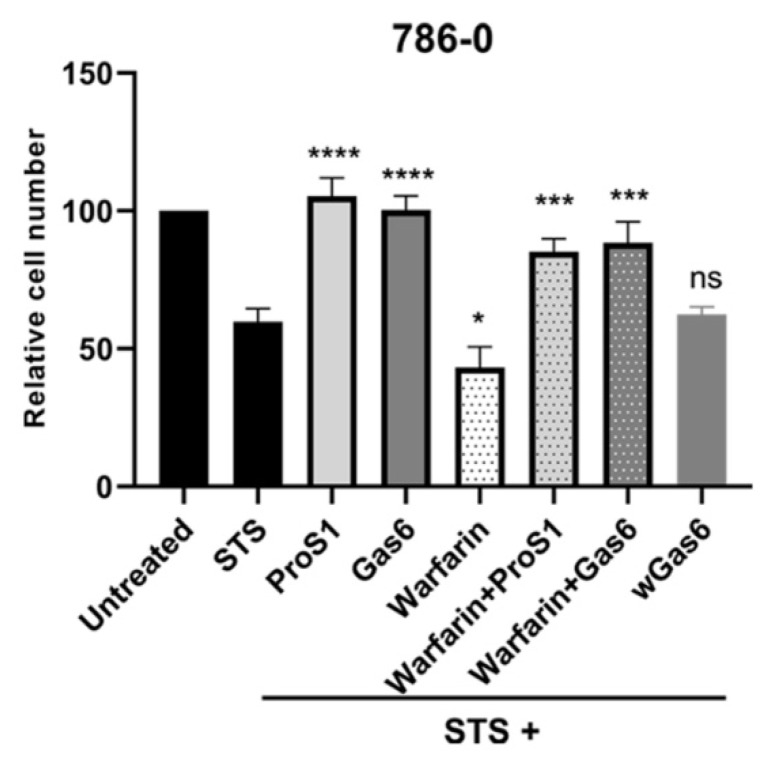
MTS assay showing the influence of vitamin K antagonism by warfarin on growth/viability of 786-0 (ProS1-producing) cells. Apoptosis was induced in 786-0 cells by staurosporine (0.1 µM), and the effects of co-incubation with exogenous TAM ligands over 20 h was measured, both in cells pre-treated or not with warfarin for 48 h. wGas6 is a fully uncarboxylated Gas6 protein used as the negative control for the vitamin K-dependence of the functionality of Gas6. Data are mean±SEM; ANOVA with Tukey’s multiple comparison *post-hoc* analysis; **** *p* < 0.0001, *** *p* < 0.001, * *p* < 0.05 and ns, not significant, versus staurosporine (STS) (n = three separate experiments).

Further experiments showed that apoptosis induced following warfarin pre-incubation of 786-0 cells (ProS1 producer) resulted in a level of cell death greater than the effect of staurosporine alone (Figure 6). However, the addition of exogenous recombinant TAM ligands ProS1 and Gas6 to the warfarin-treated (as well as untreated) cells resulted in significantly greater cell survival in both cases. In addition, a preparation of uncarboxylated Gas6 protein, which was derived from cells pre-treated with warfarin (wGas6), and which therefore would be functionally inactive [16], had no effect on cell survival (Figure 6). 

**Figure 7:**
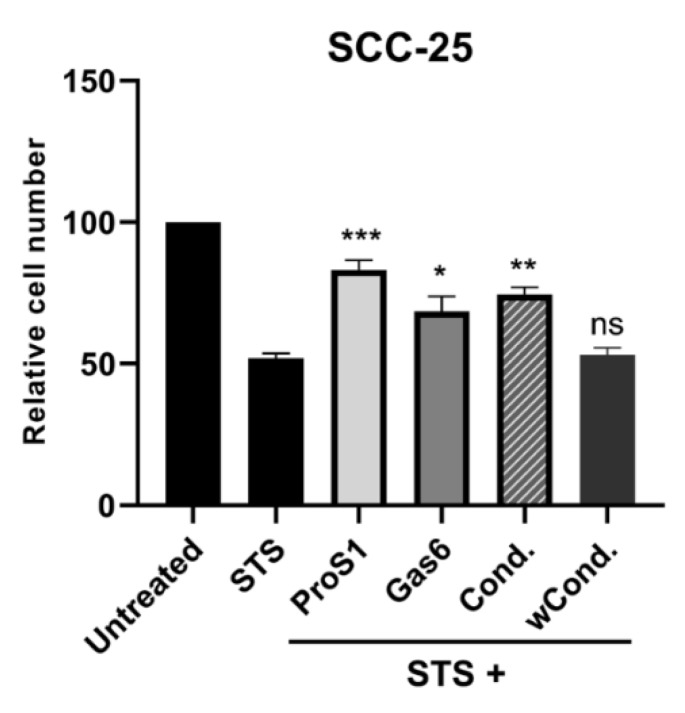
MTS assay showing the influence of vitamin K antagonism by warfarin on the functionality of natural ProS1 secreted by 786-0 cells. Apoptosis was induced by staurosporine (0.1 µM) in SCC-25 (responder) cells and the effects of co-incubation with exogenous TAM ligands for 20 h was measured, including conditioned medium from 786-0 (producer) cells collected after 48h incubation in 1% FBS media either with (wCond.) or without (Cond.) warfarin (1µg/ml). Data are mean±SEM; ANOVA with Tukey’s multiple comparison *post-hoc* analysis; *** *p* < 0.001, ** *p* < 0.01, * *p* < 0.05 and ns, not significant, versus staurosporine (STS) (n = three separate experiments).

In addition, the natural ProS1 secreted by 786-0 cells was shown to be functional only due to its vitamin K-dependent carboxylation of the Gla domain. Conditioned medium from 786-0 (producer) cells cultured in the absence of warfarin stimulated cell survival when added to SCC-25 (responder) cells undergoing the staurosporine (0.1 µM) challenge to trigger apoptosis (Figure 7). However, conditioned medium from warfarin (1 µg/mL) pre-incubated 786-0 producer cells had no such protective effect. Therefore, these results together show that ProS1 is secreted as a functional, vitamin K-dependent ligand from human cancer cells and stimulates cancer cell survival via Tyro3 activation. 

## 3. Discussion

In this study, we have identified the ProS1-Tyro3-Erk axis as a signal transduction pathway in human cancer cells distinct from the previously reported Gas6-Axl-Akt axis [5]. As the roles of both Tyro3 RTK and ProS1 in cancer cell biology remained to be characterised, the Tyro3-activating abilities of both TAM ligands were compared. Specifically, by directly comparing the effects of both ligands in cells expressing different compositions of TAM RTKs, we were able to determine that Tyro3 has versatility in its ligand sensitivity and the intracellular signalling interactions it couples to. 

We first characterised a variety of different human cancer cell lines for their expression of TAMs and TAM ligands. Tyro3 was the most ubiquitously expressed out of the three TAM RTKs, whereas the other two TAMs exhibited more discrete expression patterns. Also, the two TAM ligands, Gas6 and ProS1, showed discrete expression patterns across the cell line panel. A notable example was 786-0 kidney cancer cells, which showed high ProS1 expression but negligible Gas6 expression. Out of these cell lines, two were selected as models in which to probe Tyro3 function; one of these was SCC-25 head and neck squamous cell carcinoma cells, which express both Axl and Tyro3 but not MerTK, whilst the other, MGH-U3 bladder carcinoma cells, expresses Tyro3 as a sole TAM receptor. Our data show that ProS1 is an active and functional ligand for Tyro3 in human cancer cells, rapidly stimulating its phosphorylation as well as Erk, moreover, predominating over Gas6 as a ligand for this specific RTK. In contrast, and as is already established [11,29], Gas6 is a functional ligand for Axl RTK. Given that the SCC-25 cell line expresses both Tyro3 and Axl with negligible Mer expression and the known receptor affinity of ProS1, we deduced that the ProS1 ligand effects are via Tyro3 stimulation only. This was confirmed by the fact that Gas6 was a relatively weak ligand for Tyro3 but instead strongly stimulated Axl and Akt phosphorylation in these cells. 

As MGH-U3 cells exclusively express Tyro3 [28], we therefore used this cell line to study Tyro3 function in the absence of any hetero-interaction with other TAMs. In this setting, we observed a broadened role for Tyro3 in terms of ligand sensitivity and downstream signalling. Where Tyro3 is the only TAM receptor present, Gas6 was also an activating ligand for this RTK although weaker than ProS1. Furthermore, Tyro3 was additionally coupled to Akt signalling as observed by the fact that both TAM ligands stimulated Akt phosphorylation. However, ProS1 remained the sole Erk-activating ligand. In accordance with the signalling pathways identified, both ProS1 and Gas6 equally protected cells from apoptosis induced acutely or by prolonged serum-starvation. Therefore, these data demonstrate that Tyro3 is a significant functional RTK in cancer cells and is also responsive to ligand stimulation to activate intracellular signalling pathways, amongst which is cell survival. This role is further highlighted in tumours where Tyro3 is present as the sole TAM receptor and is sensitive to stimulation by both vitamin K-dependent TAM ligands. Furthermore, these data reveal versatility to signalling interactions for Tyro3, as illustrated by its responsiveness to Gas6 and its coupling to Akt signalling downstream (this time stimulated by both ligands); neither of these occurred in cells expressing both Tyro3 and Axl. Therefore, this specific cellular expression context and ligand responsiveness of Tyro3 indicates that it is able to present a docking interface for phosphatidylinositol 3-kinase, an event that does not occur in other cellular contexts such as in SCC-25 cells, where Tyro3 is not coupled to Akt signalling. 

Our data also reveal a greater significance for the role of ProS1 as a TAM ligand than had previously been known in extra-hepatic tissues and beyond its well-established role in regulation of blood coagulation [14,30]. The two vitamin K-dependent proteins, Gas6 and ProS1, have previously been shown to possess differential specificities and binding affinities for the three TAM receptors [11,12]. Gas6 has been shown to preferentially bind and activate Axl in the absence of PtdSer, but activates all three TAM RTKs similarly in the presence of PtdSer. In contrast, ProS1 preferentially activates Tyro3 and MerTK both in the presence and absence of PtdSer [15,31]. Furthermore, Gas6 interacts simultaneously with a contact site within each of the two tandem Ig domains of Axl, whereas only one contact site is thought to exist in Tyro3 [32]. Our data indicate that ProS1 is a stronger ligand for Tyro3 than Gas6 in a system using cultured cells where the RTKs are endogenous and functional. This is particularly clear in the MGH-U3 cells that express Tyro3 only and therefore there is no other RTK present to compete for Gas6 binding. Therefore, these data reveal a more significant role for both ProS1 and Tyro3 in cancer cell signalling than previously thought. Unusually, one study has reported stimulation of Axl by ProS1 [18]. In the present study, however, we did not observe Axl (Supplementary Figure S2A) nor Akt (Supplementary Figure S1B) stimulation by ProS1 in Axl-expressing cancer cells. In addition, by contrast, some studies have shown inhibitory effects of Gas6/ProS1 on tumour growth through inhibition of angiogenesis [33,34]. Therefore, the net effect of TAM ligands on tumourigenesis may depend on the combination of these distinct cell responses. 

In addition, we were interested to determine the functionality of endogenous ProS1 secreted by human cancer cells, and therefore, its potential as an autocrine or paracrine TAM ligand on cancer cells. As 786-0 renal carcinoma cells expressed ProS1 protein strongly, we used the conditioned medium from these cells and SCC-25 cells as responder cells to compare against the already observed effect of exogenous recombinant ProS1. Our results showed that ProS1 naturally secreted from cancer cells is functional in being able to activate Tyro3 and Erk kinase downstream to the same extent as recombinant ProS1. Furthermore, the cancer cell-derived ProS1 was shown to be active due to gamma-carboxylation in a vitamin K-dependent process, as warfarin pre-treatment of producer cells rendered the conditioned medium ineffective on Tyro3 in responder cells. Furthermore, the conditioned medium effect was confirmed to be due to ProS1 specifically through the blocking effects observed of specific ProS1 ligand traps, including soluble Tyro3 extracellular domain and anti-ProS1 neutralising antibody. Therefore, our data demonstrate that cancer cells can be a major source of functional ProS1, expressed and modified post-translationally in a vitamin K-dependent process exactly as that which exists for several vitamin K-dependent proteins in the liver. Furthermore, cancer cell-derived ProS1 is a functional activator of Tyro3 and hence can be an autocrine or paracrine supporter of tumour progression. Similar observations have been made with locally sourced Gas6 as a TAM stimulator on tumour cells [35]. 

While numerous studies have described signalling pathways downstream of Axl and MerTK and the changes following stimulation with TAM ligands [12,13], relatively few studies have directly assessed signalling pathways downstream of Tyro3, particularly in cancers. Tyro3 was early on found to be autophosphorylated upon overexpression in a ligand-independent manner, and an association with Src kinase was detected [4,26]. In bone, Gas6 was shown to stimulate mouse osteoclast function via Tyro3, involving activation of Erk [22] and Erk signalling mediated Tyro3 regulation of proliferation in breast cancer [27]. Tyro3 knockdown studies in tumour xenograft models have shown a role for Tyro3 in tumour progression, as well as the association of Tyro3 expression with poor prognosis in, amongst others, colorectal, hepatocellular, breast, and bladder cancers [28,36]. Tyro3 has also been linked to Akt signaling and cell survival regulation in e.g., hepatocellular carcinoma [25,26]. Tyro3 shRNA knockdown promoted apoptosis and decreased anchorage-independent growth in e.g. melanoma cells [37]. Inhibition studies have also revealed a role for Tyro3 in metastasis, reflected in decreased cancer cell migration and invasion [25]. Furthermore, Tyro3 has also been implicated as a mediator of resistance to anti-cancer agents [6]. The present study directly compared both TAM ligands as Tyro3 ligands in native Tyro3-expressing human cancer cell lines, the biological functionality of tumour secreted ProS1, and the diversifying capability of Tyro3 in terms of coupling to intracellular signalling. Our data has shown that, in cells where both Tyro3 and Axl are present, the discrete signalling axes ProS1-Tyro3-Erk and Gas6-Axl-Akt exist side by side. The ProS1-Tyro3 link to Erk signalling indicates the potential to regulate cell growth, whilst Gas6-Axl-Akt regulates cell survival and/or invasive signalling, as has previously been shown by ourselves and others [19,38,39]. Furthermore, in cells where Tyro3 is the sole TAM receptor and therefore gateway for all TAM ligands, we have shown that it is connected to both Erk and Akt signalling pathways and hence able to potentially regulate both cancer cell proliferation and survival respectively. Indeed, ProS1 is a more prominent TAM ligand in this context, as it was able to stimulate both Erk and Akt intracellular signalling pathways whilst Gas6 only stimulated Akt signalling where both TAM receptors were present. These novel findings demonstrate that Tyro3 is also linked to cancer cell survival as well as proliferation through the actions of ProS1, given that ProS1 only activates Tyro3 and not Axl. 

## 4. Material and Methods

### 4.1. Cell Culture

A variety of human cancer cell lines from various sources were grown in culture (Supplementary Table S1). Cells were normally cultured in “complete” medium, comprising Dulbecco’s Modified Eagle Medium (Fisher Scientific, Loughborough, UK), supplemented with 10% foetal bovine serum (FBS) (Lonza, Slough, UK), and 1% penicillin/streptomycin (Fisher Scientific). Cells were routinely maintained at 37 °C in a humidified incubator with 5% CO_2_ and were typically passaged once they reached 80% confluency, through dissociation with trypsin/EDTA (Fisher Scientific). 

### 4.2. Cell Treatments

The SCC-25 and MGH-U3 cells were first serum starved for 24h, then treated with recombinant human Gas6 developed in-house [5] and recombinant human ProS1 (7.5 nM) (Cambridge Protein Works, Cambridge, UK) for the time periods indicated. Conditioned media from the cell line 786-0, which expresses high levels of ProS1, was collected after 48h incubation in 1% FBS media either with or without warfarin (1 µg/mL). The medium was then used in stimulation experiments with SCC-25 as responder cells to test Tyro3 activation. In addition, recombinant human chimeric proteins (Axl-Fc and Dtk-Fc [referred to as Tyro3-Fc]) and ProS1 antibody (BioTechne, Abingdon, UK) were used at a final concentration of 3 μg/mL and 5 μg/mL respectively. 

### 4.3. RNA Extraction and Quantitative Real-Time Polymerase Chain Reaction

Cellular total RNA was isolated using RNeasy Mini Kit (Qiagen, Hilden, Germany) according to the manufacturer’s protocol, and purity and concentration were determined using a spectrophotometer (ND-1000; NanoDrop Technologies, Wilmington, DE, USA). cDNA was synthesised from the total RNA (High Capacity cDNA Reverse Transcription Kit, Applied Biosystems, Foster City, CA, USA) according to the manufacturer’s protocol. cDNA was used directly for PCR amplification or stored at −20 °C for later use.

Quantitative PCR (qPCR) amplification from cDNA was performed using hydrolysis probes in a 96-well plate and run on a LightCycler^®^ 96 System (Roche, Burgess Hill, UK). Reactions were assembled together with a mastermix for probes (Roche). The genes were investigated using pre-designed primers/probes (Supplementary Table S2). For each reaction, the *GAPDH* gene was utilised as an endogenous control (reference) gene. The amplification conditions included 95 °C for 15 s (1 cycle) followed by 60 °C for 1 min (45 cycles). qPCR amplification data were analysed using the 2^−ΔCt^ method, as previously described [5]. 

### 4.4. SDS–PAGE and Western Blotting

For cell lysis, the media was aspirated from cells, which were washed twice in ice-cold phosphate-buffered saline (PBS; Fisher Scientific). Cells were lysed in ice-cold RIPA buffer (150 mM NaCl, 1% Triton X-100, 0.5% sodium deoxycholate, 0.1% SDS, 50 mM Tris pH 8.0) supplemented with a cocktail of protease and phosphatase inhibitors (Fisher Scientific). Cells in lysis buffer were agitated on a shaker for 40 min at 4 °C, and the removed lysates were clarified by centrifugation at 21500× *g* for 10 min at 4 °C. The supernatant was transferred into new microfuge tubes and stored at −20 °C until required. 

Cell lysates were subjected to sodium dodecyl sulphate polyacrylamide gel electrophoresis (SDS-PAGE). The separated proteins were transferred by a wet transfer method onto an activated polyvinylidene fluoride membrane (Millipore, Nottingham, UK). Membranes were incubated for 1 h at room temperature in blocking buffer, which was either Tris-buffered saline-Tween 0.1% (TBS-T; Fisher Scientific) containing 3% non-fat dry milk, or otherwise containing 3% bovine serum albumin (BSA; Fisher Scientific) if probing for phosphorylated proteins. After blocking, membranes were incubated with primary antibodies diluted in appropriate blocking buffer overnight at 4 °C, washed (3 × 5 min) with TBS-T, and then incubated with appropriate horseradish peroxidase (HRP)-conjugated secondary antibody diluted in an appropriate blocking buffer for 2 h at room temperature. Following washing with TBS-T (3 × 5min), membranes were incubated with an enhanced chemiluminescence (ECL) development reagent (Luminata Forte; Millipore) for 3 min and visualised with chemiluminescence CCD camera (ImageQuant LAS 500, GE Healthcare Life Sciences, Buckinghamshire, UK). The software *ImageJ* was used for densitometric quantification of Western blot band intensities [40]. 

The primary antibodies recognising human proteins (and dilutions) used were: Axl (C-20) (goat polyclonal; 1:1,000 Santa Cruz, Dallas, TX, USA), Tyro3 (rabbit monoclonal; 1:1,000; Cell Signalling Technology (CST), London, UK ), MerTK (B-10) (mouse monoclonal; 1:1,000; Santa Cruz), phospho-Axl (rabbit polyclonal; 1:500; R&D systems), phospho-Akt 1/2/3 (rabbit polyclonal; 1:1,000; Santa Cruz), phospho-Tyro3 (rabbit polyclonal anti-Sky/MerTK; 1:1,000; Sigma, Gillingham, UK), β-Actin (rabbit polyclonal; 1:5,000) (CST) and GAPDH (C-9) (mouse monoclonal 1:1000; Santa Cruz). Secondary antibodies used were donkey anti-rabbit HRP (1:2,000; Dako, Cambridge, UK), anti-goat HRP (1:5,000) and anti-mouse HRP (1:5,000) (Promega, Southampton, UK). 

### 4.5. Cell Survival/Growth Assay

The effects of various treatments on cell survival/growth were determined by measuring the reduction of [3-(4,5-dimethylthiazol-2-yl)-5-(3-carboxymethoxyphenyl)-2-(4-sulfophenyl)-2H tetrazolium] (MTS) compound (CellTiter 96 Aqueous, Promega) in the presence of phenazine methosulphate (PMS) (Sigma). Cells were seeded in 96-well plates and incubated overnight, prior to indicated treatments for various periods, after which MTS (0.4 μM) was added to cells together with PMS (0.3 nM) and incubated further for 2 h, and absorbance was measured at 492 nm using a spectrophotometric microplate reader (Synergy; BioTek, Potton, UK). 

### 4.6. Flow Cytometry and Apoptosis Assay

Cells in plates were treated with exogenous ligands Gas6 and ProS1 for 2 h before staurosporine (0.1 µM) was added to trigger apoptosis for a further 20 h. Following treatments, the cells were centrifuged at 400× *g* for 5 min and resuspended in Annexin V binding buffer and incubated with Annexin V-FITC and propidium iodide (PI; 50µg/mL) for 15 min at room temperature in the dark. Then, flow cytometry was used to analyse Annexin V-FITC binding at the relevant wavelengths for FITC (FL1) and PI (FL2) signal detection according to the manufacturer’s protocol (Abcam, Cambridge, UK). The software *FlowJo-V10* was used for analysis. 

### 4.7. Statistical Analysis

All data are expressed as mean±SEM and were obtained from a minimum of three independent experiments, each constituting multiple replicates per condition as specified in the figure legends. Quantitative data were analysed by Analysis of Variance (ANOVA) with post-hoc Tukey test for multiple comparisons with one control group or multiple time points per treatment, or paired *t*-test for pairwise comparisons of the control with treatment. Statistical analyses of data and their graphical representations were performed using Prism software (GraphPad Software Inc, San Diego, CA). The level of statistical significance is indicated in the figures and accompanying legends, with *p* < 0.05 considered as statistically significant. 

## 5. Conclusions

In conclusion, we have shown that ProS1 is the most prominent functional ligand for Tyro3 RTK in human cancer cells and that it is linked to the Erk signalling pathway, distinct from the more well-characterised Gas6-Axl-Akt signalling axis [5,41]. Furthermore, cancer cells can secrete a ProS1 molecule that is fully functional due to a local vitamin K-dependent post-translational process. Moreover, there is versatility in Tyro3 signalling interactions with second messengers through its capability of coupling to cell proliferation and survival and invasion pathways, thus promoting tumour progression profoundly. 

These results provide novel insights into the mechanisms of action of Tyro3 as a tumorigenic RTK in human cancers. Moreover, they have clearly identified ProS1 as a tumour-derived, activating ligand of Tyro3 beyond its role in blood coagulation regulation. Further studies can reveal the molecular mechanisms that underlie the specificity of TAM-TAM ligand interactions, as well as whether homo- or hetero-dimerisation takes place to provide additionally diversified TAM signalling in cancer. These new insights should enable a more precise and effective approach to therapeutic targeting of TAM signalling in cancers where they play major roles in tumour growth, progression or chemoresistance. 

## Supplementary Materials

The following are available online at https://www.mdpi.com/2072-6694/11/12/1843/s1, Figure S1: Effect of ProS1 and Gas6 stimulation on Tyro3 phosphorylation and signalling in six human cancer cell lines; Figure S2. Effect of ProS1 and Gas6 stimulation on Axl phosphorylation and signalling in three human cancer cell lines; Figure S3. Representative dot plot diagrams obtained by flow cytometry with Annexin V-FITC/PI double-staining of SCC-25 (A) and MGH-U3 (B) cells; Figure S4. Confirmation that staurosporine does not inhibit Tyro3 kinase activity and its activation by TAM ligands; Figure S5. MTS cell viability assay showing the anti-apoptotic influence effects of ProS1 and Gas6 to also occur when apoptosis is induced by an alternative agent, cisplatin; Figure S6. MTS cell viability assay showing that blockade of Erk signalling prevents the anti-apoptotic effect of ProS1; Figure S7. Verification of changes in levels of phosphorylated Erk (pErk) and Akt (pAkt) proteins in relation to total Erk and Akt protein levels in western blots; Table S1. Ten human cancer cell lines were analysed for expression of the TAM receptors and their ligands Gas6 and ProS1; Table S2: Primers used in qRT-PCR; Whole blot figures.
