# Supplementary material for: Tumour-Secreted Protein S (ProS1) Activates a Tyro3-Erk Signalling Axis and Protects Cancer Cells from Apoptosis
Source: Cancers (Basel). 2019 Nov 22;11(12):1843. doi: 10.3390/cancers11121843 (PMC6966665; doi:10.3390/cancers11121843)
Supplement: Supplementary file 1 [file cancers-11-01843-s001.pdf]

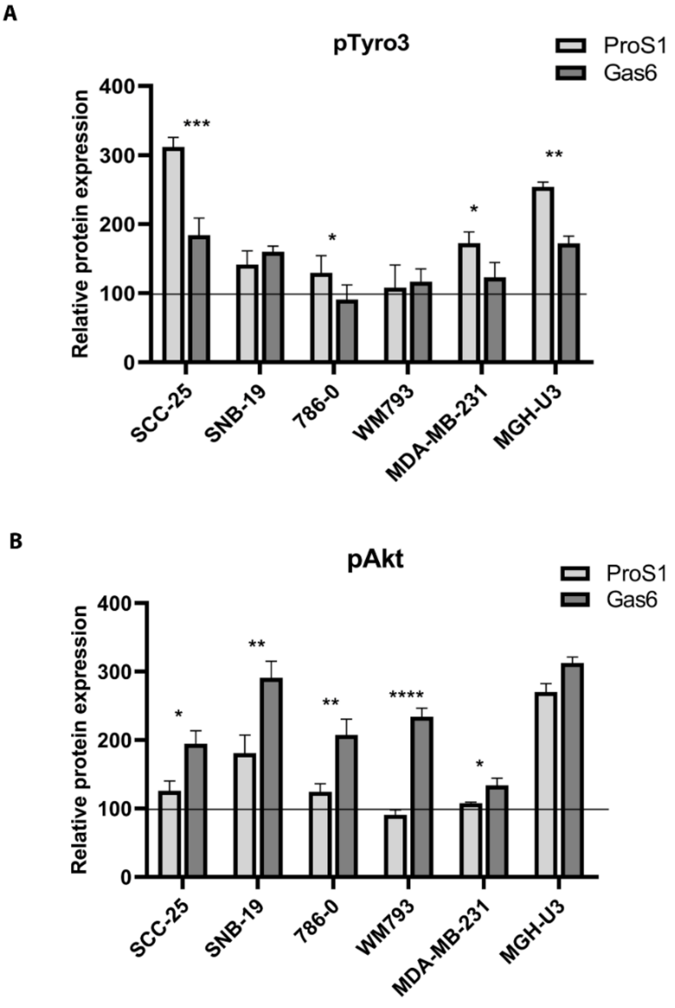

Figure S1. Effect of ProS1 and Gas6 stimulation on Tyro3 phosphorylation and signalling in six human cancer cell lines. Quantitative Western blot data showing Tyro3 phosphorylation (pTyro3) (**A**) and Akt phosphorylation (pAkt) (**B**) by Gas6 (5.7 nM) and ProS1 (7.5 nM); untreated cells marked by the horizontal bar at 100% as a negative control. Data are mean±SEM protein expression normalized against loading control protein β-actin; comparison between ProS1 and Gas6 in each cell line using Student's unpaired two-tailed *t-test*; **** $p < 0.0001$, ** $p < 0.01$, * $p < 0.05$ and ns, not significant (n = 3 separate experiments (n = 7 for SCC-25 cells)).

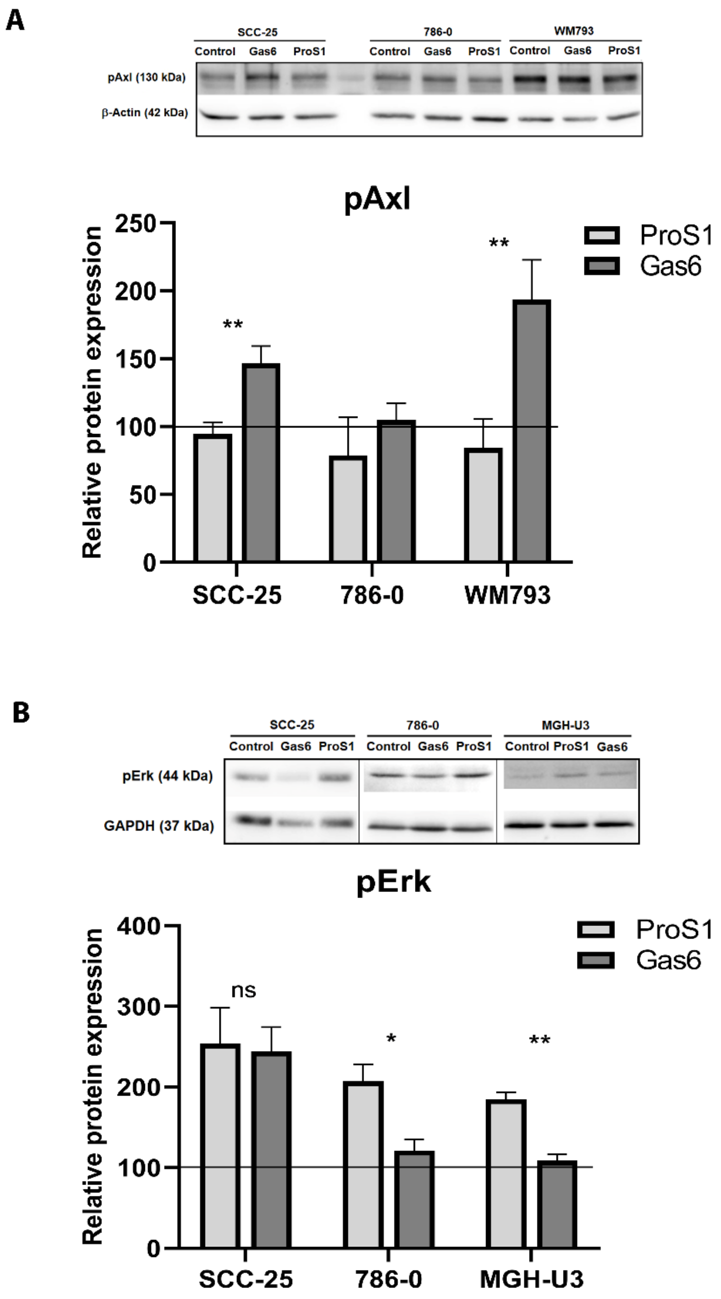

Figure S2. Effect of ProS1 and Gas6 stimulation on Axl phosphorylation and signalling in three human cancer cell lines. Quantitative Western blot data showing Axl phosphorylation (pAxl) (**A**) and Erk phosphorylation (pErk) (**B**) by Gas6 (5.7 nM) and ProS1 (7.5 nM); untreated cells marked by the horizontal bar at 100% as a negative control. Data are mean ± SEM protein expression normalized against loading control protein β-actin; comparison between ProS1 and Gas6 in each cell line using Student's unpaired two-tailed *t-test*; ** $p < 0.01$, * $p < 0.05$ and ns, not significant (n = minimum 3 separate experiments).

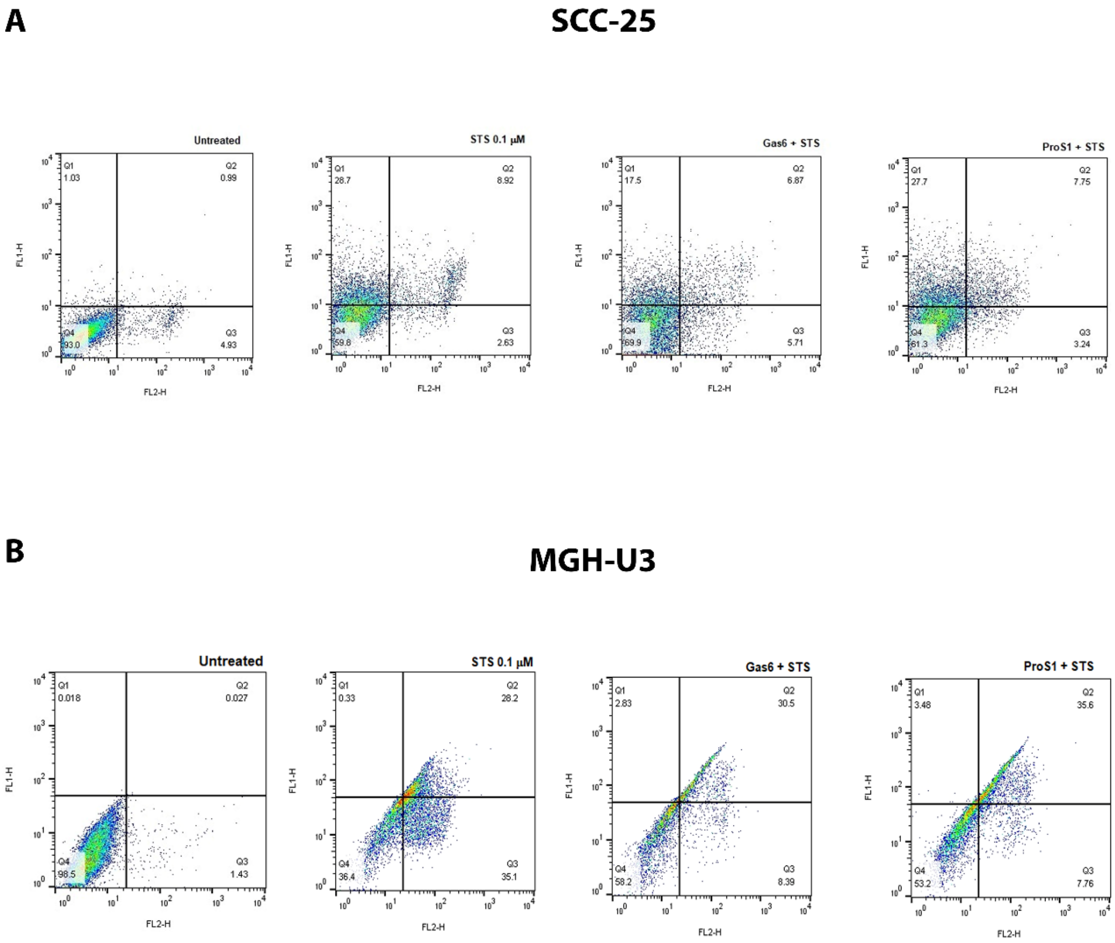

Figure S3. Apoptosis assay represented by dot plot diagrams obtained by flow cytometry with Annexin V-FITC/PI double-staining of SCC-25 (**A**) and MGH-U3 (**B**) cells. Lower left quadrant A^-/PI^- corresponds to living cells, lower right quadrant A^+/PI^- corresponds to early apoptotic cells, upper right quadrant A+/PI+ corresponds to late apoptotic cells and upper left quadrant A^-/PI^+ corresponds to necrotic cells.

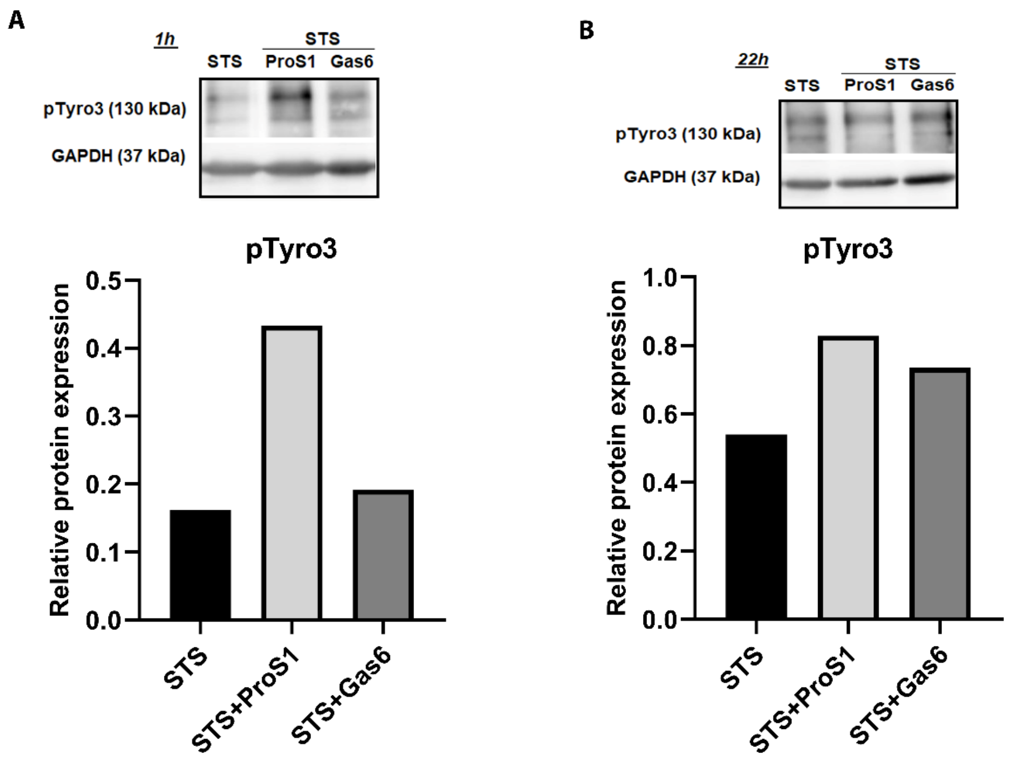

Figure S4. Confirmation that staurosporine does not inhibit Tyro3 kinase activity and its activation by TAM ligands. Apoptosis was induced in SCC-25 cells by incubation with staurosporine (0.1 μM) for 1h (**A**) and for 22 h (**B**), followed by co-incubation with exogenous ProS1 (7.5 nM) and Gas6 (5.7 nM). Cell lysates underwent Western blotting to detect phospho-Tyro3 protein. Quantitative analysis by densitometric analysis of bands is shown graphically below each blot image.

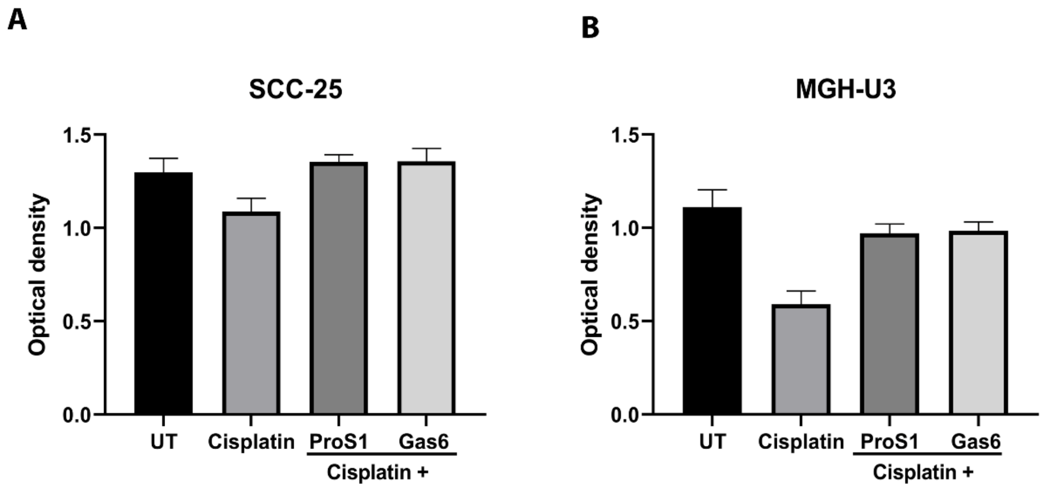

Figure S5. MTS cell viability assay showing the anti-apoptotic effects of ProS1 and Gas6 to also occur when apoptosis is induced by an alternative agent, cisplatin. SCC-25 (**A**) and MGH-U3 (**B**) cells were incubated for 20 h in the presence of cisplatin (10 μM), in the absence and presence of Gas6 or ProS1, added 1h previously. Data are mean ± SD from a representative experiment with quadruplicate wells per treatment.

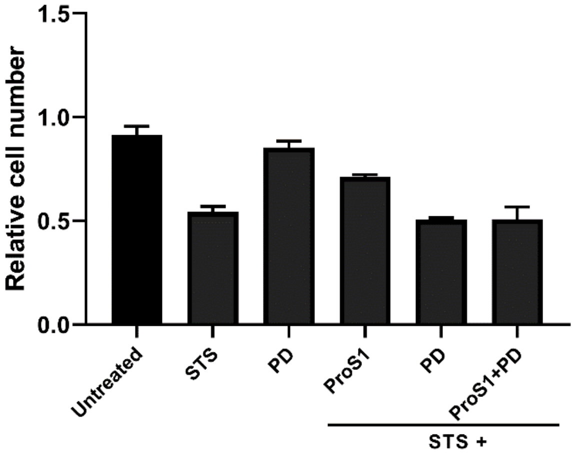

Figure S6. MTS cell viability assay showing that blockade of Erk signalling prevents the anti-apoptotic effect of ProS1. MGH-U3 cells were incubated for 20h in the presence of staurosporine (STS) (0.1 μM), in the absence or presence of MEK inhibitor PD0325901 (PD; 2 μM), with ProS1 (7.5 nM) added 1h previously to some wells. Co-incubation of STS and PD0325901 with cells did not further increase the apoptotic effect of STS. However, PD0325901 blocked the cell viability-increasing (protective) effect of ProS1. Data are mean ± SD from a representative experiment with quadruplicate wells per treatment.

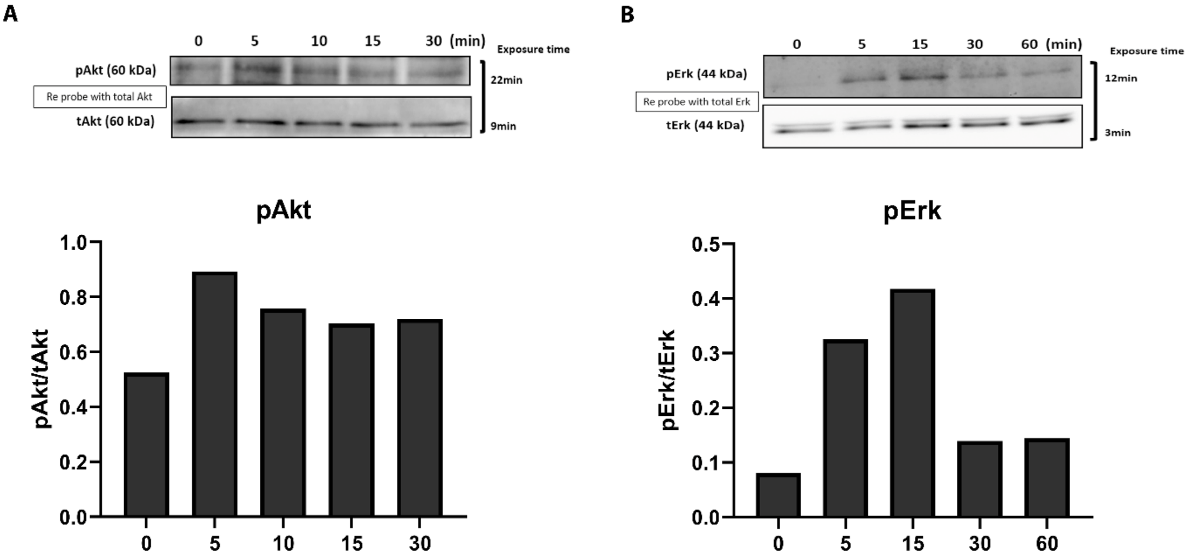

Figure S7. Verification of changes in levels of phosphorylated Erk (pErk) and Akt (pAkt) proteins in relation to total Erk and Akt protein levels in Western blots. Cells were stimulated with ProS1 ligand over a 30-min time course, and Western blots of lysates were probed first with anti-pErk and anti-pAkt antibodies, followed by re-probing the same blots with anti-total Erk (tErk) and anti-total Akt (tAkt) antibodies, respectively. Blots show that ProS1 increases both pAkt (MGH-U3; **A**) and pErk (SCC-25; **B**) levels over the time course relative to levels of corresponding total proteins. Following band densitometry, normalized phosphoprotein levels (phospho:total) are shown graphically below the blot images. Blot exposure times are also displayed, showing shorter exposure times for the total protein blots.

Table S1. Ten human cancer cell lines were analysed for expression of the TAM receptors and their ligands Gas6 and ProS1.

Human Cell line	Type	Source
**SCC-25**	Head and neck squamous cell carcinoma	ATCC
**SNB-19**	Glioblastoma	DSMZ
**786-0**	Renal carcinoma	ATCC
**MDA-MB-231 (MDA)**	Breast cancer	ATCC
**WM793**	Melanoma	ATCC
**O14**	Melanoma	Sigma
**WM9**	Melanoma	Rockland, Limerick, PA, USA
**RT112**	Bladder cell carcinoma	ECACC
**MGH-U3**	Bladder cell carcinoma	Gift from Prof. Margaret Knowles, University of Leeds
**SH-SY5Y**	Neuroblastoma	ECACC

Table S2. Primers used in qRT-PCR.

Primer	NCBI Accession number	Source
AXL	NM_021913(2)	IDT, Leuven, Belgium
TYRO3	NM_006293(1)	IDT
MERTK	NM_006343(1)	IDT
GAS6	NM_000820(3)	IDT
PROS1	NM_000313(1)	IDT
GAPDH	NM_001686.3/ NM_001256799.1	Life technologies, Warrington, UK

Whole Blots

Membranes from each gel were cut at different molecular weights in order to probe for different proteins of interest in their respective mol wt ranges. Membranes were then incubated with the corresponding antibodies, and were exposed for development for the appropriate lengths of time for each protein. Chemiluminescence images of uncropped membranes are shown below, with band densitometry values shown above or below each band lane of that representative blot. Molecular weight markers are not always visible in some membranes at the exposures used; the arrows indicate their positions.

Blots for Figure 1A:

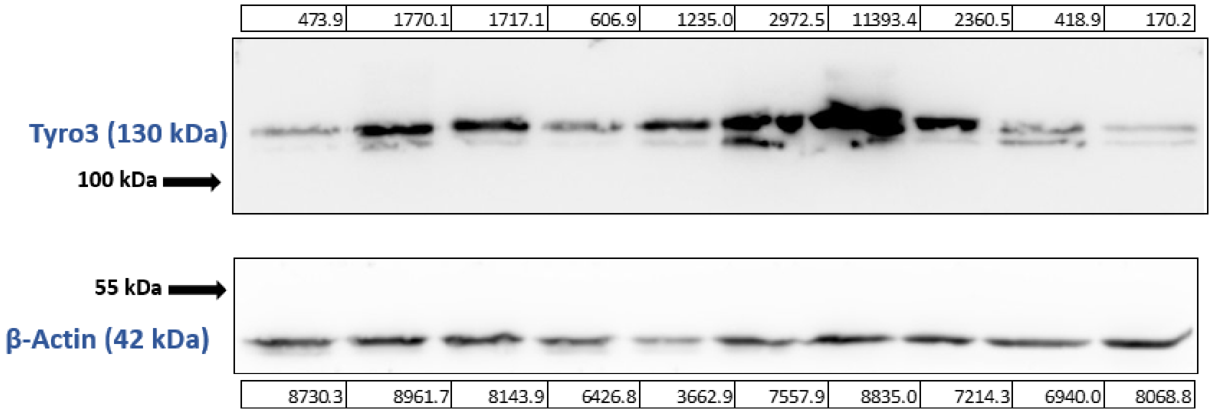

Membranes from same gel showing Tyro3 and β-actin.

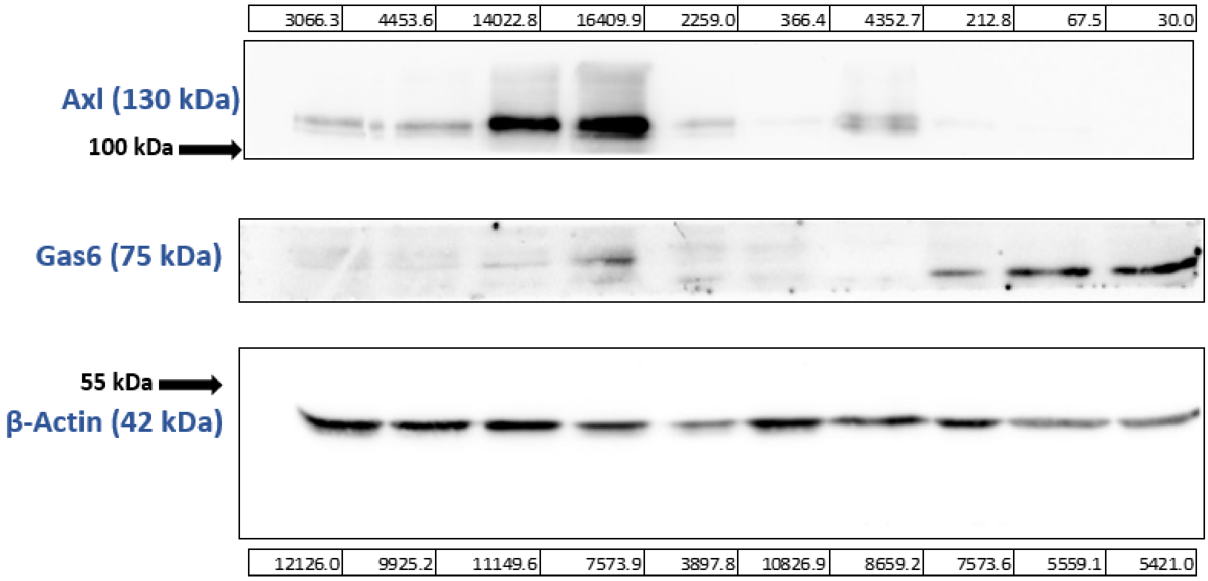

Membranes from same gel showing Axl, Gas6 and β-actin.

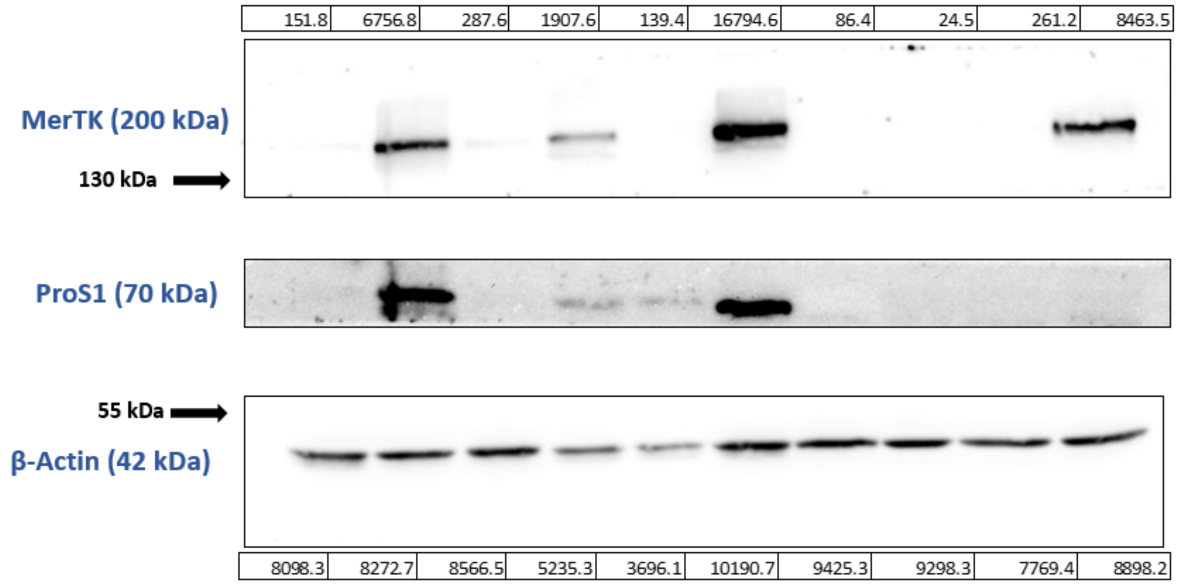

Membranes from same gel showing MerTK, ProS1 and β-actin.

Blots for Figure 2:

A

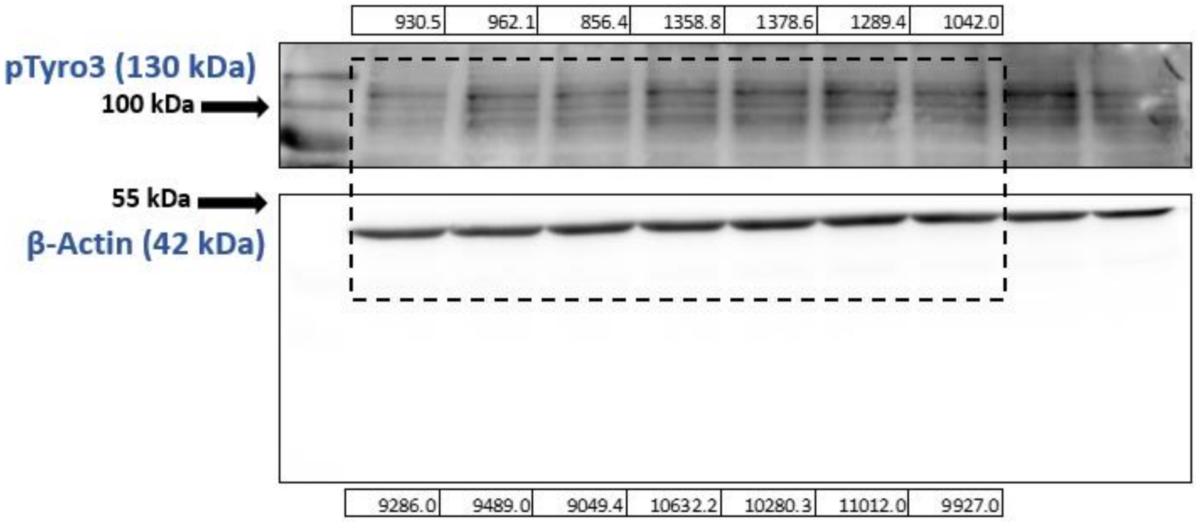

Membranes from same gel showing pTyro3 and β-actin. Box with dotted line edges outlines the part of the blot displayed in the figure of the paper that includes the samples relevant to the experiment.

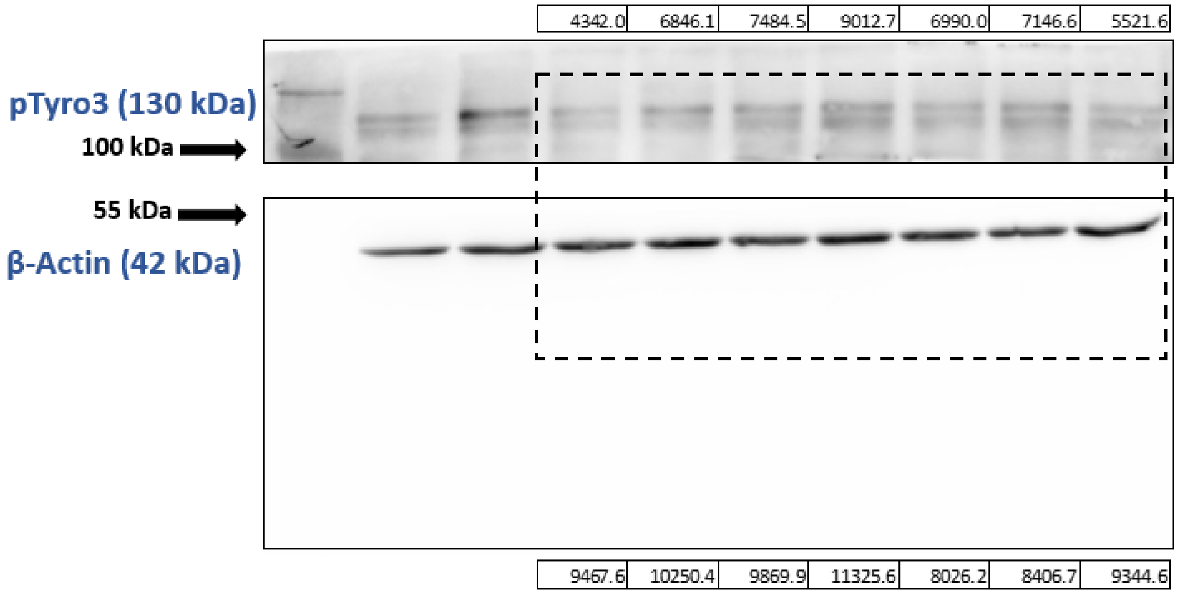

Membranes from same gel showing pTyro3 and β-actin. Box with dotted line edges outlines the part of the blot displayed in the figure of the paper that includes the samples relevant to the experiment.

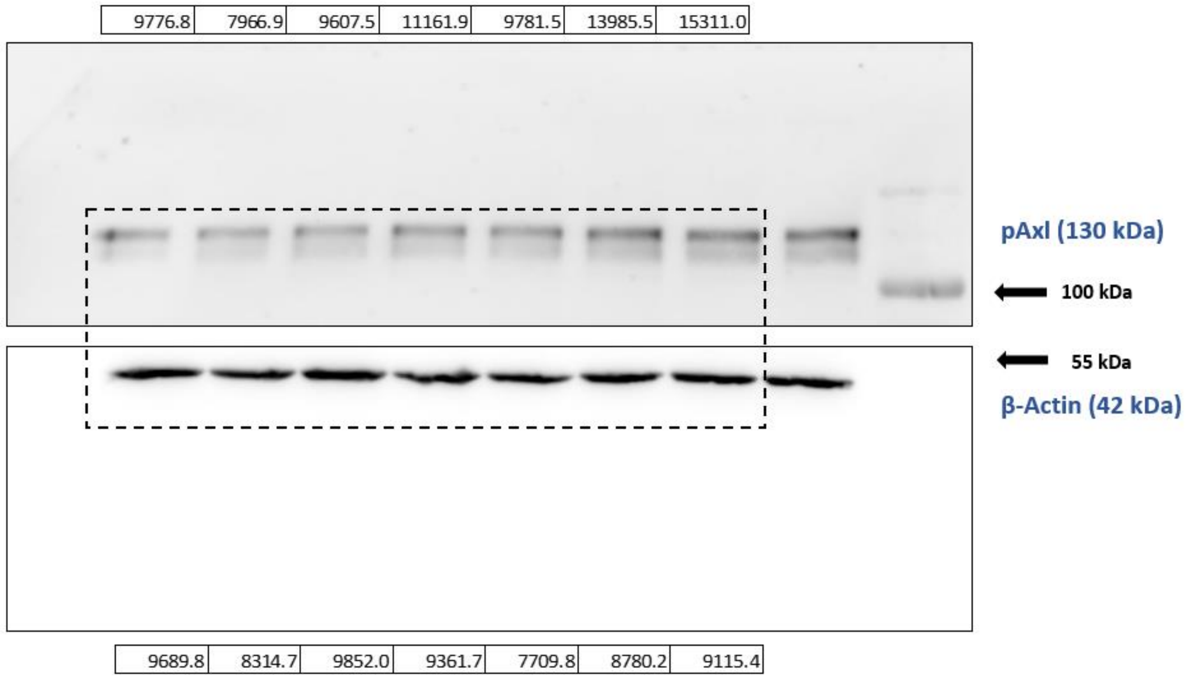

Membranes from same gel showing pAxl and β-actin. Box with dotted line edges outlines the part of the blot displayed in the figure of the paper that includes the samples relevant to the experiment. In each membrane, the part between 70 and 55 was used to detect pAkt (not presented).

B

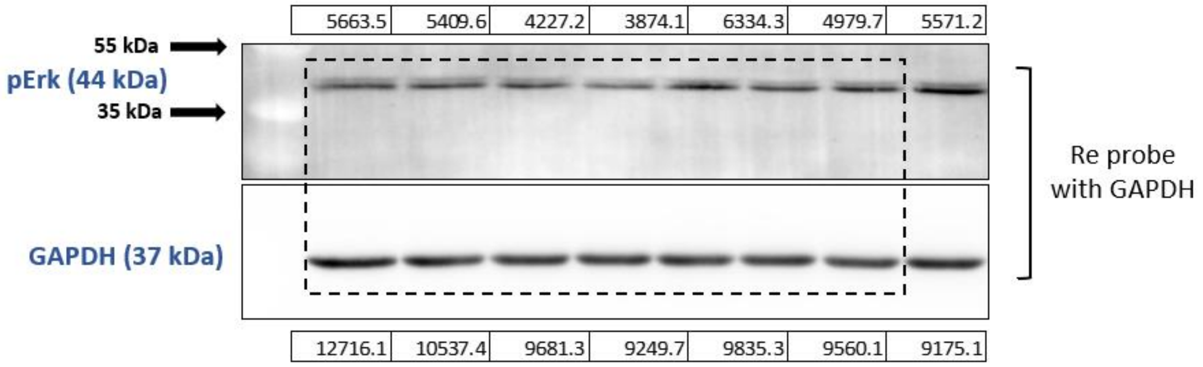

Membrane from same gel showing pErk and GAPDH. The membrane (2 images of the same part of the membrane) was first probed with anti-pErk antibody, developed, then re-probed (without stripping) with anti-GAPDH antibody, which at the shorter exposure time did not reveal the original pErk bands. Box with dotted line edges outlines the part of the blot displayed in the figure of the paper that includes the samples relevant to the experiment.

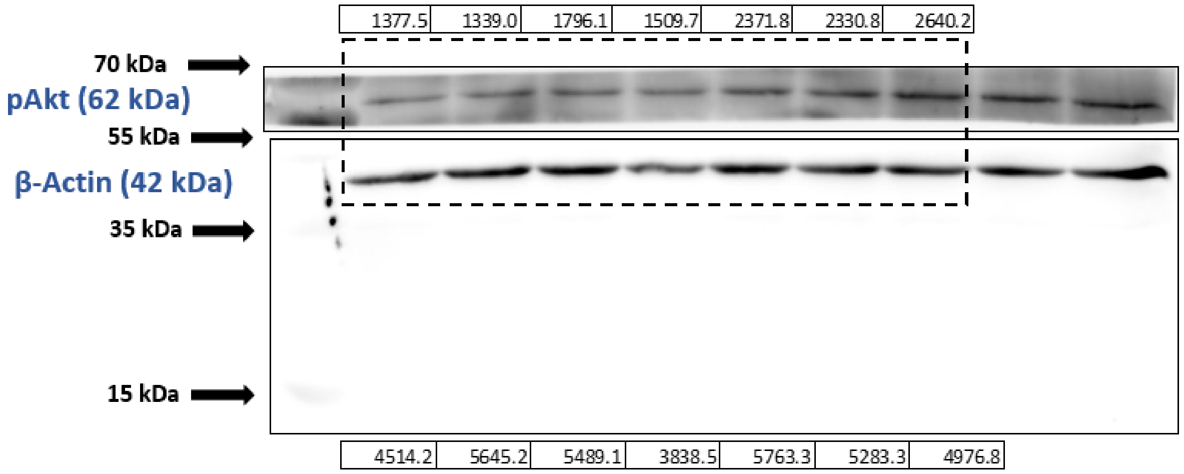

Membranes from same gel showing pAkt and β-actin. Box with dotted line edges outlines the part of the blot displayed in the figure of the paper that includes the samples relevant to the experiment.

Blots for Figure 3:

A

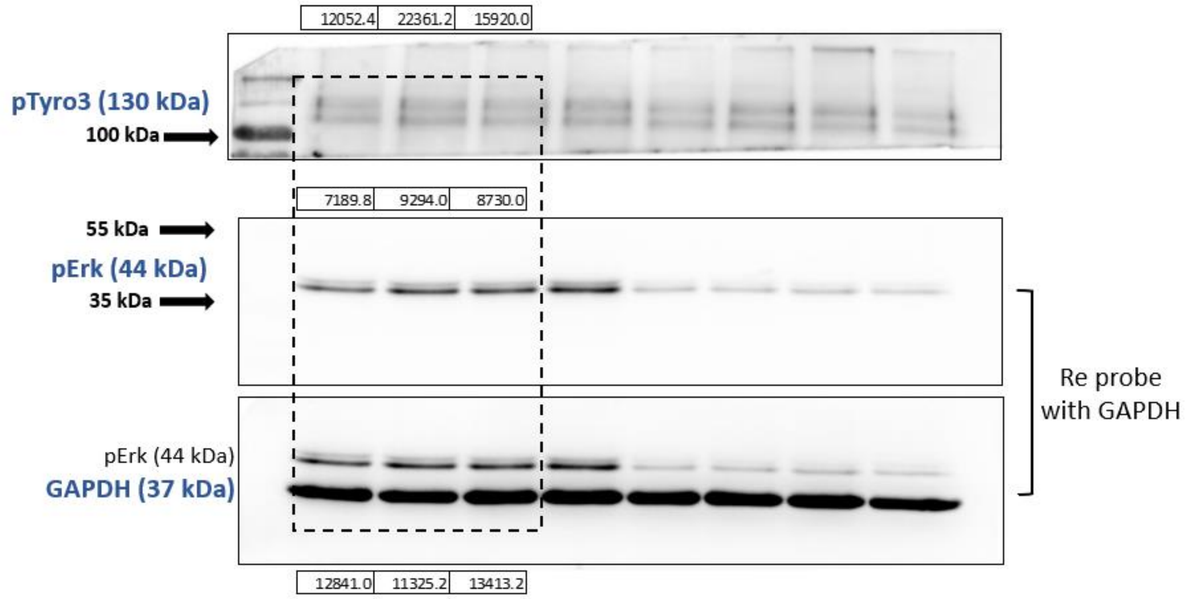

Membranes from same gel showing pTyro3, pErk and GAPDH. The lower membrane (2 images of the same part of the membrane) was first probed with anti-pErk antibody, developed, then re-probed (without stripping) with anti-GAPDH antibody, which required a shorter exposure time to reveal the GAPDH bands, whilst also maintaining visibility of the first probe pErk bands. Box with dotted line edges outlines the part of the blot displayed in the figure of the paper that includes the samples relevant to the experiment.

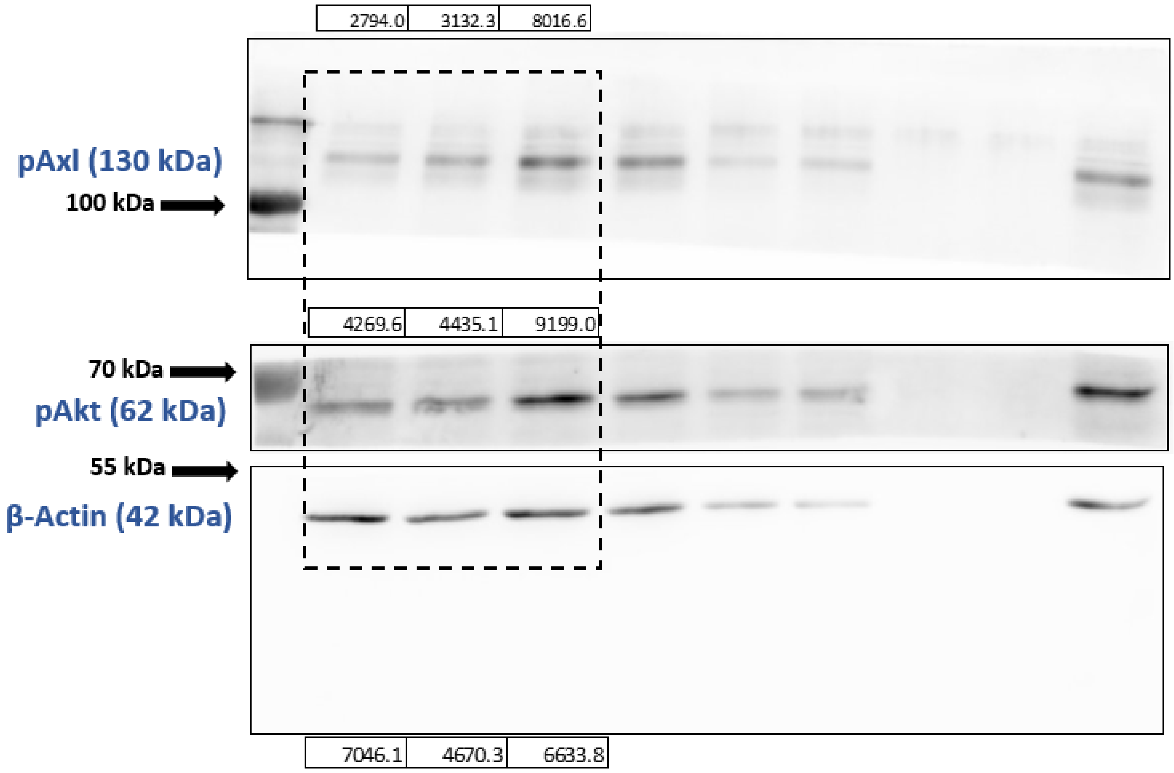

Membranes from same gel showing pAxl, pAkt and β-actin. Box with dotted line edges outlines the part of the blot displayed in the figure of the paper that includes the samples relevant to the experiment.

B

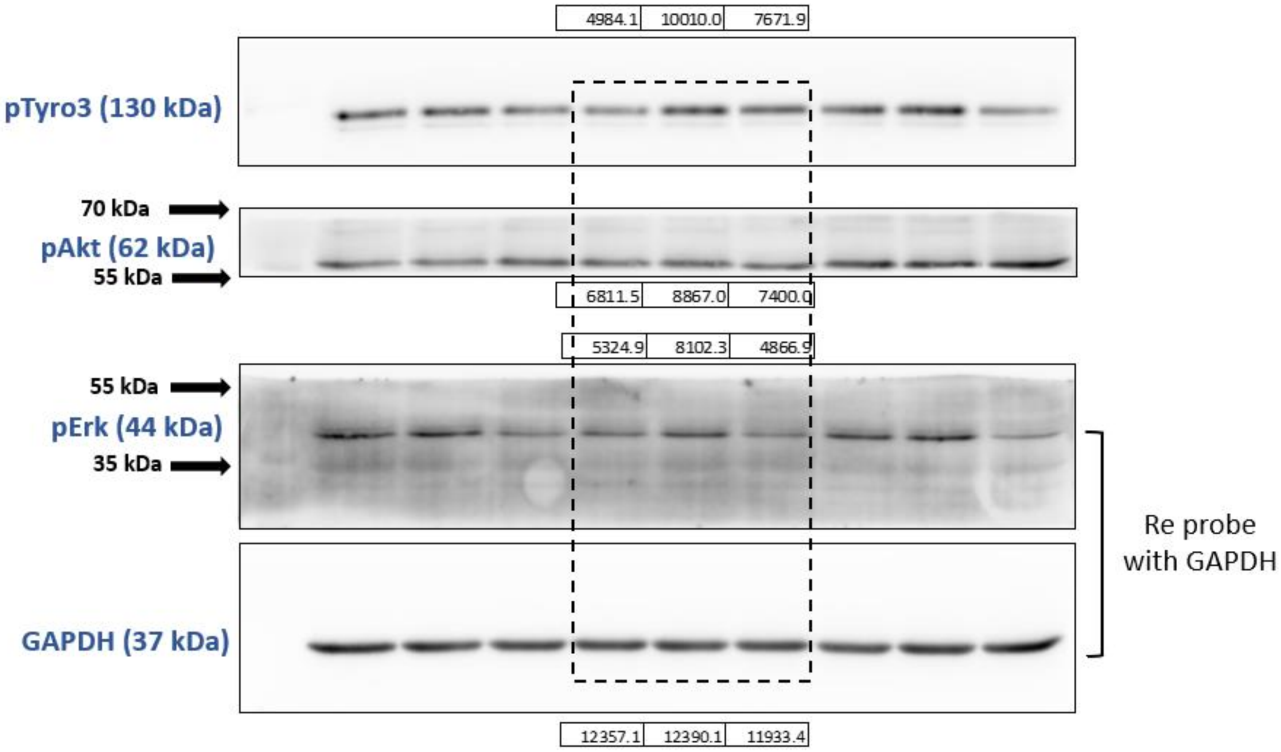

Membranes from same gel showing pTyro3, pAkt, pErk and GAPDH. The lowest 2 membranes (2 images of the same part of the membrane) was first probed with anti-pErk antibody, developed, then re-probed (without stripping) with anti-GAPDH antibody, which at the shorter exposure time did not reveal the original pErk bands. Box with dotted line edges outlines the part of the blot displayed in the figure of the paper that includes the samples relevant to the experiment.

Blots for Figure 4:

A

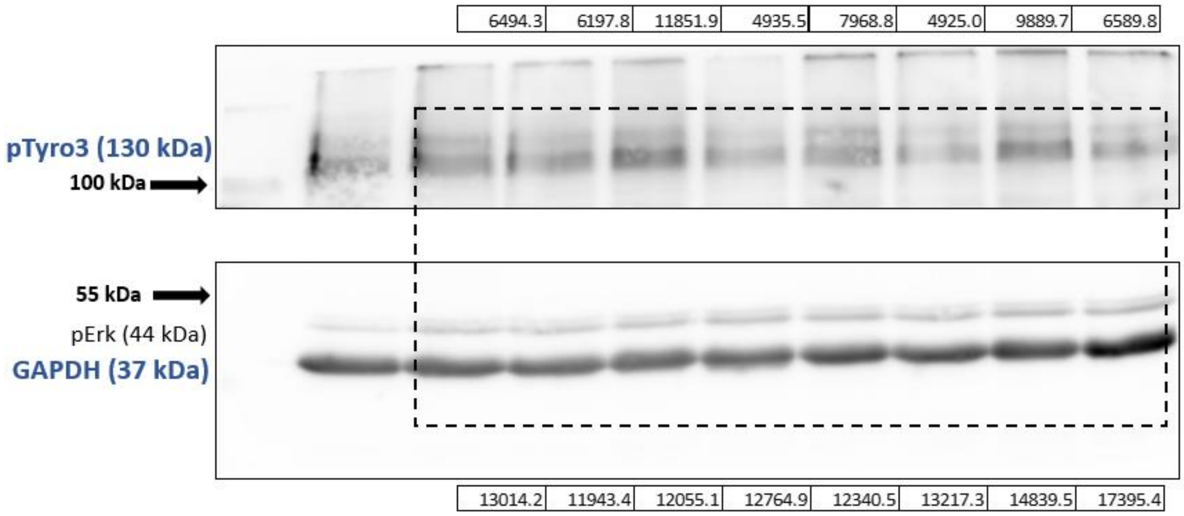

Membranes from same gel showing pTyro3 and GAPDH. Box with dotted line edges outlines the part of the blot displayed in the figure of the paper that includes the samples relevant to the experiment.

B

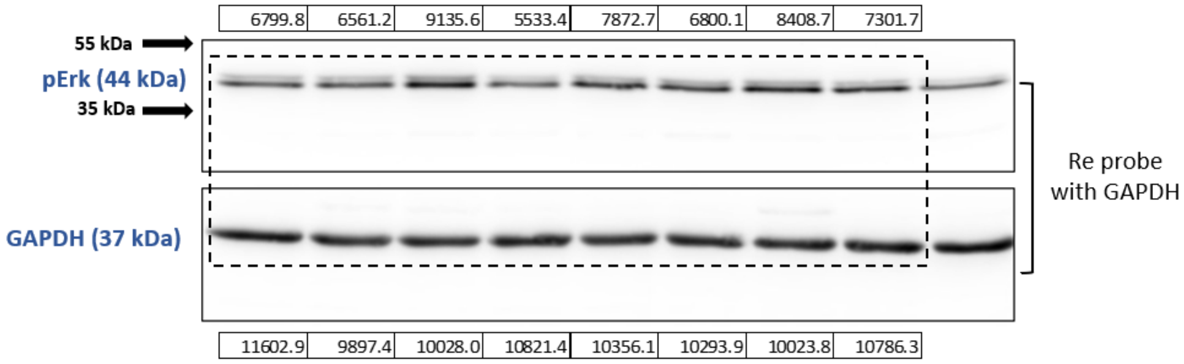

Membrane from gel showing pErk and GAPDH (2 images of the same membrane). The membrane was first probed with anti-pErk antibody, developed, then re-probed (without stripping) with anti-GAPDH antibody, which at the shorter exposure time did not reveal the original pErk bands. Box with dotted line edges outlines the part of the blot displayed in the figure of the paper that includes the samples relevant to the experiment.

Blots for Figure S2:

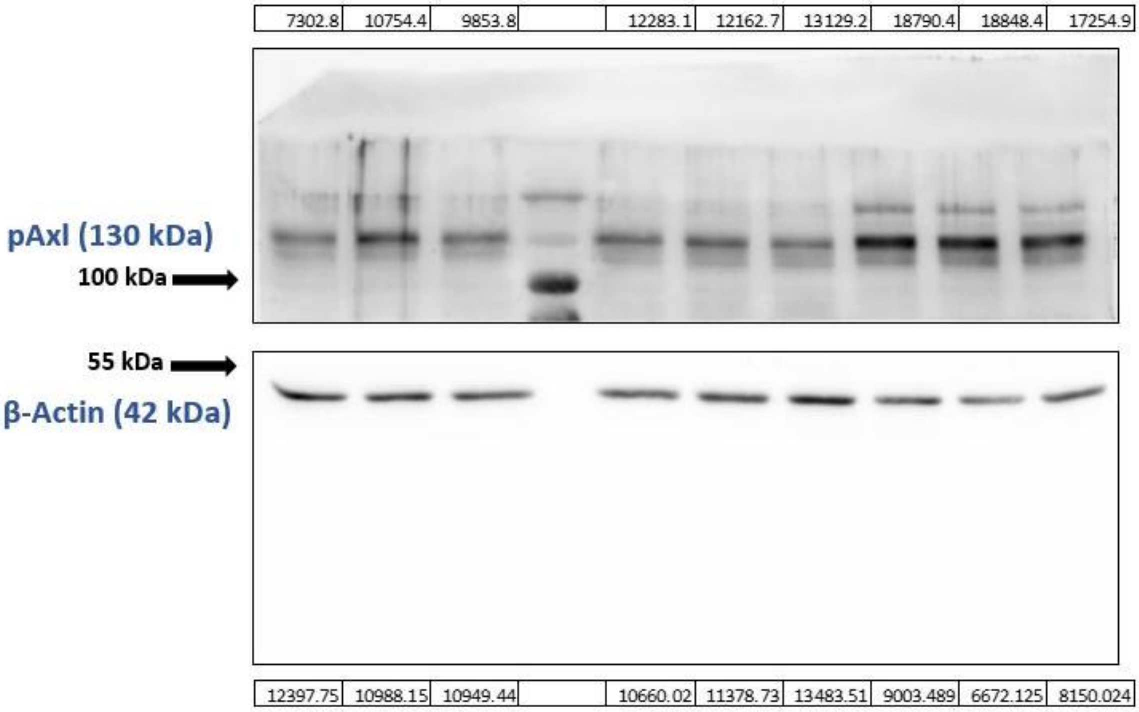

Membranes from same gel showing pAxl (upper part) and β-actin (lower part) following probing with the relevant antibodies, and developed at different optimal exposure times.

Blots for Figure S4:

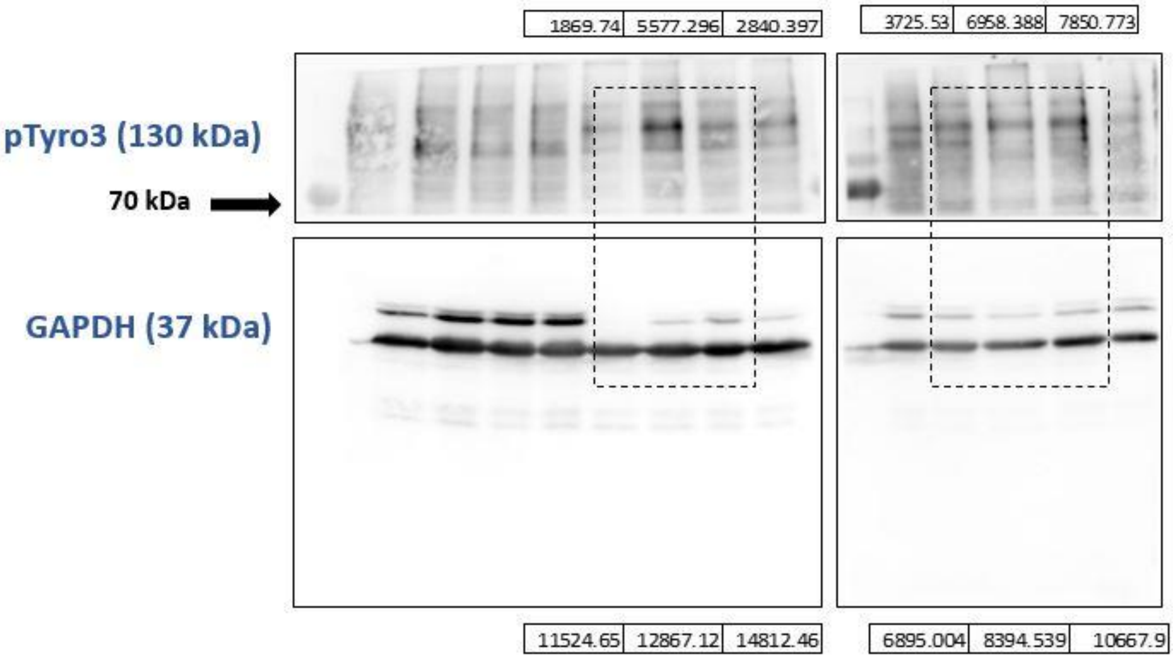

Membranes from same gel showing pTyro3 and GAPDH. Box with dotted line edges outlines the part of the blot displayed in the figure of the paper that includes the samples relevant to the experiment. Note that the bands visible above the GAPDH bands were pre-probed with anti-pErk

antibody, developed, then re-probed (without stripping) with anti-GAPDH antibody – however, the pErk probe bands were not relevant to this experiment.

Blots for Figure S7:

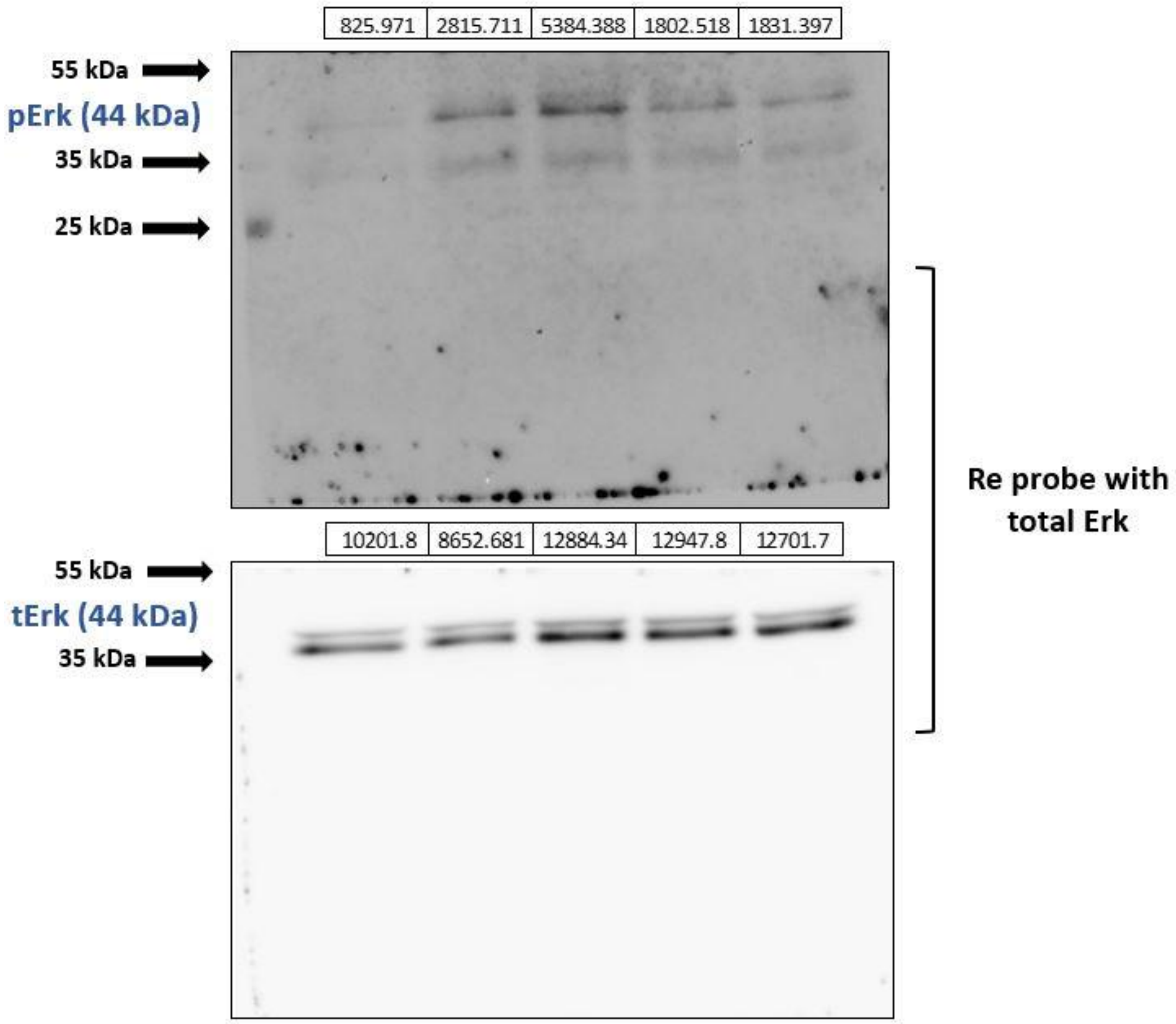

Membrane from same gel showing pErk and tErk (2 images of the same membrane). The membrane was first probed with anti-pErk antibody (exposure time 12 min), then re-probed (without stripping) with anti-tErk antibody, which at the shorter exposure time 3 min did not reveal the original pErk bands.

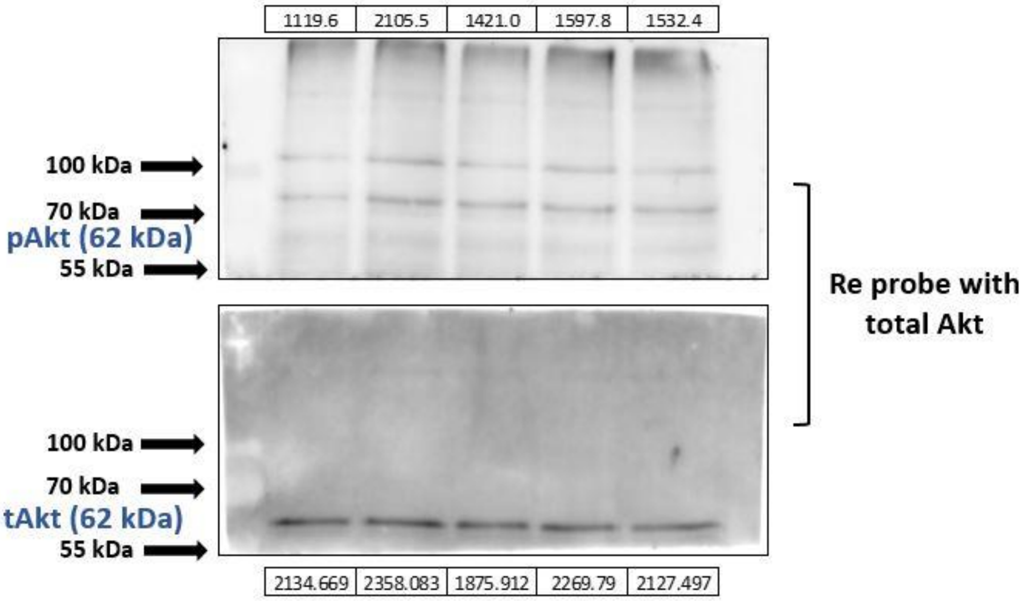

Membrane from same gel showing pAkt and total Akt (tAkt) (2 images of the same membrane). The membrane was first probed with anti-pAkt antibody (upper; exposure time 22 min), then re-probed (without stripping) with anti-tAkt antibody (lower), which at the shorter exposure time 9 min did not reveal the original pAkt bands.
